# Epidermal and ECM Damage Following Pinch Injury Restricts Dendrite Regeneration in *Drosophila*

**DOI:** 10.1523/ENEURO.0058-26.2026

**Published:** 2026-09-25

**Authors:** Mia A. Brantley, Avantika Pandiyan, Annie C. Danh, Sydney E. Prange, Dario S. Rimicci, Katherine L. Thompson-Peer

**Affiliations:** ^1^Department of Developmental and Cell Biology, University of California, Irvine, California 92697; ^2^Center for the Neurobiology of Learning and Memory, Irvine, California 92697; ^3^Sue and Bill Gross Stem Cell Research Center, Irvine, California 92697

**Keywords:** dendrite injury, dendrite regeneration, directed regrowth, *Drosophila*, ECM injury, wound repair

## Abstract

Neuronal dendrites can be injured by a number of insults, but the cellular mechanism by which dendrites respond to tissue injury and undergo repair is poorly understood. Much of the field's progress has evaluated dendrite regeneration following laser injury. While precise, laser injury does not accurately model the real-world damage to surrounding tissue that would accompany neuronal injury. Here, we modify a pinch injury technique to injure both the dendrites and their surrounding tissues in both female and male *Drosophila melanogaster* larvae to better model what is observed following real-world neuronal trauma. We refined this technique such that only half of a sensory neuron's dendrites are injured, leaving the other half uninjured. Our data indicate that both dynamic and stable dendritic arbors regrow dendrites in response to pinch injury, and neurons primarily engage in compensatory regeneration whereby new branches are added on the uninjured half of the arbor. Comparing the regenerative response following pinch injury versus laser injury revealed that dendrites preferentially regrow into areas where the surrounding tissue was left intact and not into areas where the surrounding tissue was damaged by pinch. These results prompted us to evaluate the damage sustained to surrounding tissues. In examining non-neuronal tissues after pinch injury, we found damage to the epidermis and ECM, but not glia. We also observed a robust immune response in the pinched hemisegment. We conclude that the surrounding tissue damage combined with a sustained immune response creates a nonpermissive environment for dendrite regeneration following pinch injury.

## Graphical abstract

**Figure:**
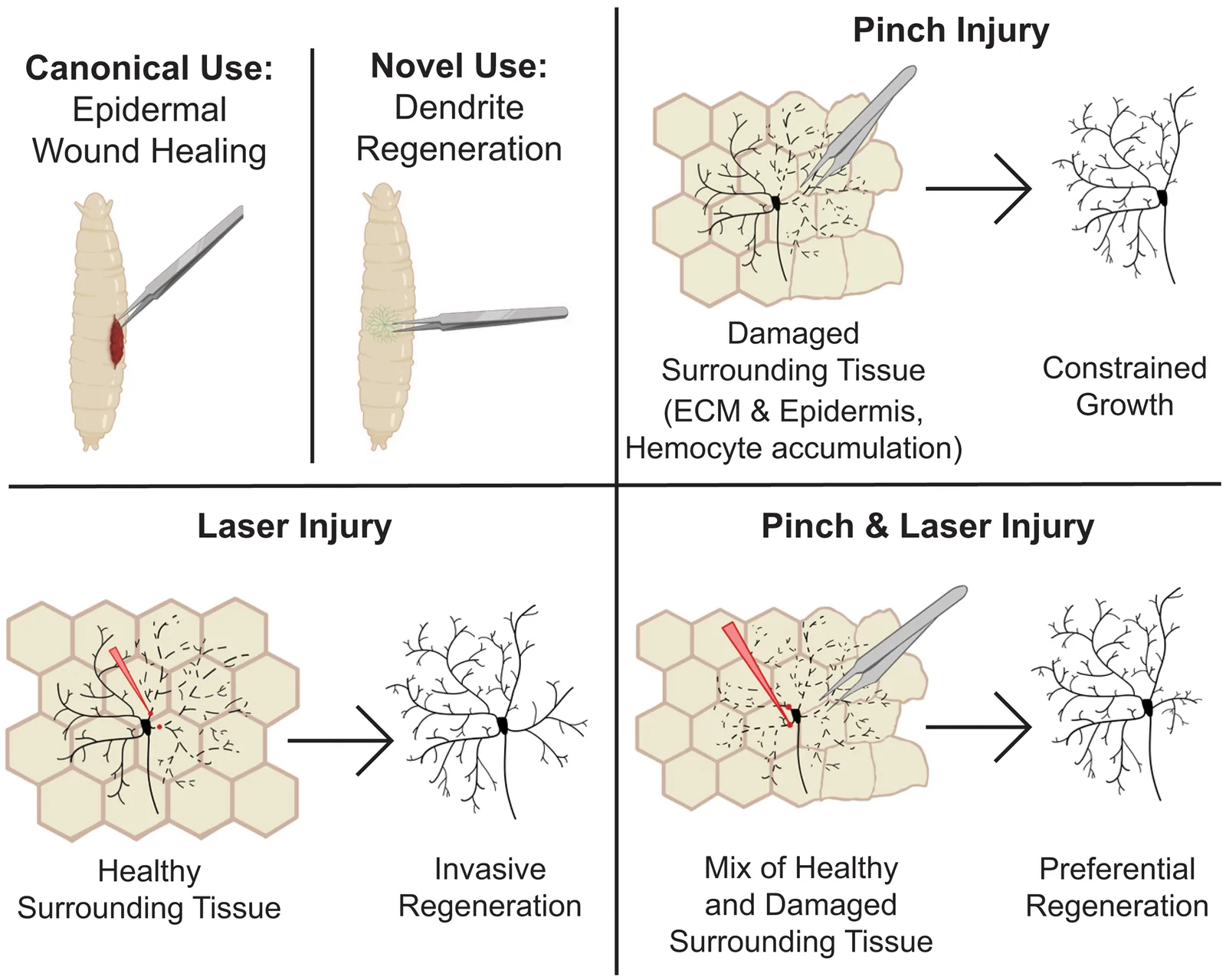
Visual Abstract

## Significance Statement

Neuronal dendrites are injured in clinical conditions, such as stroke, traumatic brain injury, and neonatal hypoxia. Dendrites also degenerate in the early stages of a number of neurodegenerative diseases. The role of surrounding tissues in dendrite regeneration is poorly characterized, especially considering that neuronal injury is typically accompanied by broad tissue damage. Our data evaluate dendrite regeneration following an injury that better mirrors real-world conditions and demonstrates that broad tissue damage diminishes a neuron's capacity to regenerate its dendrites. Our findings show that neurons preferentially regrow into intact, undamaged tissue environments, addressing a large gap in the field's knowledge: how damage to the surrounding tissue limits neuron regeneration after injury.

## Introduction

Neurons are the main signal transducers of the nervous system. They are composed of two distinct structures: dendrites that receive signals and axons that send those signals. Unfortunately, dendrites can be injured by a number of insults like stroke, hypoxia, traumatic brain injury (TBI), and neurodegenerative disease. In response to this damage, dendrites are capable of regeneration. Following total dendrite laser resection, *Drosophila melanogaster* peripheral nervous system (PNS) dendrites can regenerate the same number of branches as age-matched uninjured control neurons (Song et al., 2012; Stone et al., 2014; Thompson-Peer et al., 2016). Similarly, *Caenorhabditis elegans* PNS PVD neurons can regenerate their dendrites via plasma membrane fusion (Oren-Suissa et al., 2017; Brar et al., 2022). Zebrafish spinal cord motor neurons regenerate their dendrites 5–7 d post laser microdissection, demonstrating that central nervous system (CNS) dendrites are also capable of regeneration (Stone et al., 2022). Albeit limited, dendrite regeneration in the mammalian CNS has been observed immediately following laser microsurgery in the mouse brain and spinal cord (Zhao et al., 2017) and following brain prick injury in the adult mouse cerebral cortex (Paveliev et al., 2016).

To investigate the mechanisms of dendrite regeneration, we use the dendritic arborization (da) sensory neurons of the *Drosophila* PNS (Grueber et al., 2002). These da neurons are grouped by class (I–IV) and innervate the epidermis of the larval body wall. Development of the da neural system depends upon permissive extracellular environments and signals from surrounding cell types and tissues. For example, an epidermally derived microRNA, *bantam*, regulates the scaling growth of class IV da neurons (Parrish et al., 2009; Jiang et al., 2014). Proper space-filling of class IV da neurons requires a permissive signal created by heparan sulfate proteoglycans (HSPGs) on the epidermal cell surface (Poe et al., 2017). The extracellular matrix (ECM) also plays a critical role in dendrite development in other systems (Dansie and Ethell, 2011; Levy et al., 2014; Long and Huttner, 2019; Heiman and Bülow, 2024).

In addition to normal development, recent studies hint that extracellular signals also regulate da dendrite regeneration. Epidermally derived *bantam* signaling restricts dendrite regeneration in class IV ddaC neurons (Song et al., 2012). Regenerated dendrites exhibit decreased ECM adherence, which is mediated by integrins and results in failure of self-avoidance mechanisms (Thompson-Peer et al., 2016). Further, dendrite regeneration in adult flies is constrained by ECM remodeling due to epidermally expressed matrix metalloproteinase 2 activity (DeVault et al., 2018). Alterations in ECM attachment may cause many of the morphological differences that newly regenerated dendrites display, such as decreased area coverage (Thompson-Peer et al., 2016).

Dendrite regeneration is a novel and growing field, but it has not been studied as extensively as axon regeneration, considering how traditional surgical injury approaches are not suitable for dendrite injury (Peterson and Benowitz, 2018; Varier et al., 2022; Hertzler and Rolls, 2024; Vaughn and Lee, 2024). Recent advancements in microscopy have overcome these challenges to studying dendrite regeneration (Song et al., 2012; Stone et al., 2014; Thompson-Peer et al., 2016; DeVault et al., 2018; Nye et al., 2020; Hertzler et al., 2023; Duarte et al., 2024; Hertzler and Rolls, 2024; Prange et al., 2024; Hwu et al., 2025), but these studies utilized a laser injury method, which is slow, has a low throughput, and does not accurately model real-world neuronal damage. Given that the role of extracellular cues in dendrite regeneration needs deeper discovery, how well an injury method models real-world trauma warrants consideration.

A more accurate means of modeling real-world injury has been demonstrated by developing an epithelial pinch wound assay to study wound healing and reepithelization in *Drosophila* larvae (Galko and Krasnow, 2004; Stevens and Page-McCaw, 2012; Burra et al., 2013; Tsai et al., 2017). An RNAi screen utilizing this pinch wounding assay identified genes involved in proper wound healing (Lesch et al., 2010), and it has been adapted to evaluate ECM wound healing (Ramos-Lewis et al., 2018).

Here, we have established a pinch injury technique to injure the da neurons and surrounding tissues. This injury method damaged class IV and class I da neurons, the epidermis, and ECM, while leaving the glia largely uninjured. Pinch injury also recruited hemocytes to the injured hemisegment. Response to this damage was stereotyped: dendrites preferentially regrew into empty territory where surrounding tissue was left intact, while surrounding tissue damage restricted dendrite regeneration. This pinch injury method was faster and more accurately modeled damage to the neuron, its dendrites, and surrounding tissue. Overall, we find that damage to the ECM and other surrounding tissues impedes dendrite regeneration.

## Methods and Materials

### Experimental model and subject details

#### Drosophila strains

*Drosophila* stocks were maintained at room temperature. The following fly strains were used in this study: Canton-S, ppk-CD4-tdGFP (second chromosome, BDSC #35842; Han et al., 2011), ppk-CD4-tdGFP (third chromosome, BDSC #35843; Han et al., 2011), Gal4^ppk^ (second chromosome, BDSC #32078; Grueber et al., 2003), UAS-CaMPARI2.L398T (second chromosome, BDSC #78319; Moeyaert et al., 2018), Gal4^2–21^ (third chromosome, BDSC #26259; Grueber et al., 2003), UAS-CD4-tdGFP (second chromosome, BDSC #35839; Han et al., 2011), Gal4^2–21^, UAS-CD4-tdTomato (third chromosome), ppk-CD4-tdTomato (second chromosome, BDSC #35844; Han et al., 2011), Gal4^A58^ (third chromosome; Galko and Krasnow, 2004), viking-GFP Protein Trap (second chromosome, BDSC #98343; Morin et al., 2001), UAS-viking-GFP (third chromosome, courtesy of Noselli Lab; Van De Bor et al., 2015), Gal4^GMR38F11^ (third chromosome, BDSC #50014; Jenett et al., 2012), UAS-Armadillo-GFP (third chromosome, BDSC #58725), armP::Armadillo-GFP (third chromosome, BDSC #8555; Orsulic and Peifer, 1996), Gal4^repo^, UAS-mRFP (third chromosome; Sepp et al., 2001), Gal4^pxn^ (third chromosome, BDSC #600223; Stramer et al., 2005), UAS-CD4-tdTomato (second chromosome, BDSC #35841; Han et al., 2011), UAS-RedStinger^6^ (third chromosome, BDSC #8547; Barolo et al., 2004).

A complete list of fly stocks can be found in Table 1.

**Table 1.** Key resources

Reagent or resource	Source	Identifier
Chemicals		
DuPont Molykote High Vacuum Grease	Electron Microscopy Sciences	Catalog #60705
Glycerol ReagentPlus, ≥99.0% (GC)	Sigma Aldrich	CAS #56-81-5
Isoflurane	Piramal, Midwest Vet Supply	Item #193.33165.3
GeneMate LE Quick Dissolve Agarose	VWR	Catalog #E3119-500
Experimental models: organisms/strains		
*Drosophila*: Canton-S	Lab Stock	FlyBase_FBsn0000274
*Drosophila*: WeeP304 (tau-GFP)	Melissa Rolls (Stone et al., 2008)	FlyBase_FBal0249389
*Drosophila*: w[1118]; P{w[+mC]=ppk-CD4-tdGFP}1b	BDSC	RRID:BDSC_35842, FlyBase_FBtp0068012
Drosophila:; ppk-Gal4^VK34 / CyOw; ppk-CD4-tdGFP / Tm6B	Lab Stock	
*Drosophila*: w[1118]; P{y[+t7.7] w[+mC]=UAS-CaMPARI2.L398T}su(Hw)attP5	BDSC	RRID:BDSC_78319, FlyBase_FBti0199488
*Drosophila*: w[*]; betaTub60D[Pin-1]/CyO; P{?GawB}221w-	BDSC	RRID:BDSC_26259, FlyBase_FBti0114336
*Drosophila*: y[1] w[*]; P{w[+mC]=UAS-CD4-tdGFP}8M2	BDSC	RRID:BDSC_35839, FlyBase_FBti0143426
*Drosophila*:; + ; Gal4^2-21, UAS-CD4-tdTomato / Tm6B	Lab Stock	
*Drosophila*: w[1118]; P{w[+mC]=ppk-CD4-tdTom}4a	BDSC	RRID:BDSC_35844, FlyBase_FBti0143430
*Drosophila*:;; A58-Gal4	Lab Stock	FlyBase_FBal0181674
*Drosophila*: w[1118]; P{y[+t7.7] w[+mC]=GMR38F11-GAL4}attP2	BDSC	RRID:BDSC_50014, FlyBase_FBti0135407
*Drosophila*: y[1] w[*]; P{w[+mC]=PTT-un}vkg[G00454]	BDSC	RRID:BDSC_98343, FlyBase_FBti0153267
*Drosophila*: +; sp / CyO; repo-Gal4, UAS-mRFP / TM6B	Lab Stock	
*Drosophila*: w[*]; M{w[+mC]=UASp-arm.mGFP6}ZH-86Fb/TM6B, Tb[1]	BDSC	RRID:BDSC_58725, FlyBase_FBti0164941
*Drosophila*: w[*]; P{w[+mW.hs]=arm-GFP.P}83	BDSC	RRID:BDSC_8555, FlyBase_FBti0058765
*Drosophila*:;; UAS-Viking-GFP / Tm3, Sb	Stéphane Noselli	FlyBase_FBtp0125825
*Drosophila*: w[1118]; P{w[+mC]=Pxn-GAL4}3	BDSC	RRID:BDSC_600223, FlyBase_FBti0230342
*Drosophila*: y[1] w[*]; P{w[+mC]=UAS-CD4-tdTom}7M1	BDSC	RRID:BDSC_35841, FlyBase_FBti0143428
*Drosophila*: w[1118]; P{w[+mC]=UAS-RedStinger}6	BDSC	RRID:BDSC_8547, FlyBase_FBti0040830
Software and algorithms		
ImageJ (FIJI)	Wayne Rasband	RRID:SCR_002285 https://fiji.sc
Adobe InDesign	Adobe	RRID:SCR_021799 https://www.adobe.com/products/indesign.html
GraphPad Prism	GraphPad	RRID:SCR_002798 https://www.graphpad.com
Zen Microscopy Software	Zeiss	RRID:SCR_013672 https://www.zeiss.com/microscopy/en/products/software/zeiss-zen.html
Microsoft Excel	Microsoft	RRID:SCR_016137 https://www.microsoft.com
Other		
LSM 700 fluorescent confocal microscope	Zeiss	RRID:SCR_017377
LSM 780 fluorescent confocal microscope	Zeiss	RRID:SCR_020922
LSM 900 fluorescent confocal microscope with Airyscan 2	Zeiss	RRID:SCR_022263
LSM 980 fluorescent confocal microscope with Airyscan 2	Zeiss	RRID:SCR_025048
MAI TAI 2-photon laser	Spectra-Physics	Model EHP 1040S
Dissecting microscopes (Zeiss Stemi 2000, Nikon SMZ-10A, Olympus SZ60)	Zeiss, Nikon, Olympus	Models Stemi 2000; SMZ-10A; SZ60
Fiber-Lite Mi-150 Illuminator Series, 150 w Halogen light source (MI150 Mi 150)	Dolan-Jenner Industries	Catalog #660000391010
Eppendorf micropipettes	Thermo Fisher	SKU P-10MLR
Epredia Richard-Allan Scientific Cover Glass 22 mm × 22 mm	Thermo Fisher Scientific	Cat. # 22-050-235
Fisherbrand Premium Plain Glass Microscope Slides	Thermo Fisher Scientific	Catalog #12-544-1
LEICA MZ FLIII Fluorescent sorting microscope	LEICA	Model MZ FLIII
Super High Pressure Mercury Lamp Power Supply	Nikon	Model HB-10101AF
Fisherbrand Round Bottom Disposable Borosilicate Glass Tubes with Plain End	Thermo Fisher Scientific	Catalog #14-961-27
Parafilm M Sealing Film	Sigma-Aldrich	Catalog #HS234526B-1EA
Syringe, 10 ml, 5 ml	Thermo Fisher Scientific	Catalog #14-955-458; catalog #14-955-459
*Drosophila* food	UCI Fly Food Kitchen	N/A
Yeast, active, dry	Fisher Science Education	Catalog #S25632
Welch's 100% Grape Juice	Welch's	WEL35400
Drosophila Incubator	Powers Scientific	https://powersscientific.com/humidified-incubators/
Handheld UV lamp	Maxxeon	MXN02003

10.1523/ENEURO.0058-26.2026.t1-1 Table 1-1 **Details of All Statistical Tests** Information and details of each statistical test performed in this manuscript, including test type, group comparisons, p values, and post-hoc comparisons. Download Table 1-1, PDF file.

#### Generation of fly lines and experimental crosses

Crosses were performed in an incubator at 22.5°C and 70% humidity, and eggs were collected on a plate made of grape juice and agarose (grape plate) with yeast paste to synchronize animal age. Cross progeny larvae for experiments were kept on grape plates in an incubator at 22.5°C and 70% humidity until used for injuries. After injury, larvae were individually housed in grape plates with yeast paste and maintained at 20°C and 70% humidity until the last imaging time point (72 h after injury). Both male and female larvae were used for all experiments. Assays to assess pinch or 2p injury and subsequent regeneration in class IV ddaC neurons were performed by crossing fly lines expressing ppk-CD4-tdGFP with Canton-S flies. Assays to assess pinch injury and subsequent regeneration in class I ddaE neurons were performed by crossing fly lines expressing Gal4^2–21^ and UAS-tdTomato with Canton-S flies. Assays to assess damage to whole-body ECM were performed by crossing flies expressing ppk-CD4-tdTomato to flies expressing the viking-GFP protein trap. Assays to assess damage to A58-driven ECM were performed by crossing flies expressing ppk-CD4-tdTomato and Gal4^A58^ to flies expressing UAS-viking-GFP. In Figure 4 and Extended Data Figure 4-3, assays to assess damage to epidermal cells were performed by crossing flies expressing ppk-CD4-tdTomato and Gal4^A58^ to flies expressing UAS-Armadillo-GFP. In Figure 5 and Extended Data Figures 5-1 and 5-2*B*, assays to assess damage to epidermal cells were performed by crossing flies expressing ppk-CD4-tdTomato to flies expressing armP::Arm-GFP. In Extended Data Figure 5-2A, the assay to assess damage to epidermal cells was performed by crossing flies expressing ppk-CD4-tdTomato and Gal4^GMR38F11^ with flies expressing UAS-Armadillo-GFP. Assays to assess damage to glia were performed by crossing flies expressing Gal4^repo^ and UAS-mRFP with flies expressing ppk-CD4-tdGFP. Assays to assess the immune response following injury (Fig. 4, Extended Data Fig. 4-5) were performed by crossing flies expressing Gal4^pxn^ and UAS-CD4-tdTomato with flies expressing ppk-CD4-tdGFP. Assays to quantify hemocyte number after pinch (Extended Data Fig. 4-6) were performed by crossing flies expressing ppk-CD4-tdGFP and Gal4^pxn^ with flies expressing UAS-RedStinger. Assays to assess CaMPARI conversion in class IV ddaC neurons were performed by crossing flies expressing UAS-CaMPARI2.L398T with flies expressing Gal4^ppk^ and ppk-CD4-tdGFP. Assays to assess CaMPARI conversion in class I ddaE neurons were performed by crossing flies expressing UAS-CaMPARI2.L398T with flies expressing UAS-CD4-tdGFP and Gal4^2–21^.

### Dendrite injury assays

All injury assays were performed in live, whole-mount larvae.

#### Pinch injury assay

Please refer to the schematic in Figure 1*A* for a visual explanation of the experimental flow-through of the pinch injury assay. Please refer to Movie 1 for a recording of a pinch injury and refer to Movie 2 for a close-up movie recording of a pinch injury. To perform the pinch injury assay, groups of 5–6 age-matched 96 h after egg lay (AEL) third instar larvae were collected onto a pad of 4% agarose, and the pad was transferred to a small, empty petri dish. The small petri dish was placed into a larger petri dish for anesthetization. Larvae were anesthetized with isoflurane (∼200 µl) for 45–50 s by placing a folded-up Kimwipe moistened with a small amount of isoflurane in the larger petri dish, exposing the larvae to the isoflurane vapor. After anesthetization, the larvae, sitting on the agarose pad, were transferred out of the petri dish and visualized under a light dissecting microscope. Larval segments (thoracic and abdominal) were counted until reaching abdominal segments 4 and 5. Larvae were rolled ∼45° to expose the right half of the dorsal side of the animal for pinch injury. A pair of blunted #5 forceps was used to pinch the animal. The forceps were held such that the lateral side of the tines of the forceps were what pinched the larvae, not the tips of the forceps. One tine of the forceps was aligned with the anterior side of abdominal hemisegment 4 (A4); one tine of the forceps was aligned with the posterior side of abdominal hemisegment 5 (A5). A slight scooping motion was used to properly pick up and pinch the two hemisegments together: the anterior side of A4 meets the posterior side of A5, with the cuticle region between the forceps popping out, resembling a “muffin top.” (Please see Fig. 1*A* for a cross-sectional reference of the larval “muffin top.”) By pinching abdominal hemisegments A4 and A5 together, a pinch injury will successfully remove half of the dendritic arbor of the class IV ddaC neuron in hemisegment A4 or A5, while the dendritic arbor of the other neuron will be damaged to a lesser degree. Typically, the lesser-injured neuron is only missing one-quarter of its dendritic arbor or some high-order branches. Occasionally, two successful pinch injuries can occur, wherein both neurons in hemisegments A4 and A5 have half of their dendritic arbors removed, but this was not the typical result observed following a pinch injury. The pinch was held with minimal pressure for 10 s and then released. For CaMPARI experiments, larvae were exposed to 20 s of UV light immediately after pinching. Following pinch injury, individual larvae were then housed in grape plates with yeast paste and maintained at 20°C and 70% humidity.

**Movie 1.** Movie of pinch injury method. This movie shows the positioning of the third-instar *Drosophila* larvae in preparation for pinching. It also shows the alignment of the two tines of the forceps with abdominal hemisegments 4 and 5, along with the slight “scooping” motion required to pinch the two hemisegments together. [View online]

**Movie 2.** Close-up movie of pinch injury method. This movie shows a close-up version of the pinch injury technique as shown in Movie 1. [View online]

The following information details some common issues that result when the pinch injury is not performed properly along with some troubleshooting tips to help ensure survival of the larvae and reproducibility of the pinch injury. With respect to the amount of pressure to exert during pinch injury, a “minimal” amount of pressure should be exerted. “Minimal” pressure should be enough to close the forceps but not pop the larvae. “Minimal” pressure should feel as if any subtle relaxation of the finger muscles/release of pressure would allow the tines of the forceps to separate. If too much pressure is exerted during pinch injury, a scab may form by the 24 h AI timepoint. This scab will appear reddish brown in color and frequently forms a ring-like structure on the side of the larvae that was injured by pinch. Formation of a scab will make mounting more difficult because the epidermis and cuticle are weak where the scab has formed, and the larvae may pop. Formation of a scab will also make imaging more difficult, because it is not transparent. If larvae are younger than 96 h after egg lay (AEL), they may be too small, and the force of the scooping motion and pressure exerted by pinch may pop them. During pinch injury, if the larvae are left in a puddle of water, they will be too wet and slippery to be grabbed well for pinching. Conversely, if the larvae are too dry, the cuticle may stick to the forceps, and the larvae may be ripped apart when the tines of the forceps are separated. With respect to anesthetization with isoflurane, if the larvae are completely limp and fold in half when picked up following anesthetization, they have been exposed to too much isoflurane and will most likely not survive injury. At the 24 h AI timepoint, if the larvae are dead, white, and opaque, that is another likely indication that the larvae were exposed to isoflurane for too long. Additionally, if the larvae at the 24 h AI timepoint are dead with a splotchy brown coloration, that is a likely indication that too much force was exerted during pinch.

#### Two-photon injury assay

Larvae were immobilized for mounting by being sandwiched between an agarose pad and coverslip held together with vacuum grease (Thompson-Peer et al., 2016; Duarte et al., 2024; Prange et al., 2024; Hwu et al., 2025). Glycerol was used as the mounting media. Two-photon (2p) injury assays were performed with the Spectra-Physics Mai Tai two-photon tunable laser mounted on the Zeiss LSM980 confocal microscope. Neurons were imaged using 488 nm green or 561 nm red lasers and injured using the bleaching function with the 2p laser at 820 or 860 nm. Exposure to the laser lasted for ∼3 s. For the balding injury assay, the laser was focused on primary dendrite branches near the cell body to remove all branches with the fewest cuts possible without damaging the cell body. For the half-2p injury assay, the laser was focused on primary or secondary dendrite branches to remove half of the branches in the dendritic arbor with the fewest cuts possible. After laser injury, individual larvae were housed in grape plates with yeast paste and maintained at 20°C and 70% humidity. Successful balding injury was assessed by observing dendrite blebbing immediately after injury, comparing the 24 h AI images to the before injury images to assess if the arbors look different, and evaluating territory coverage of new arbors at 24 h AI.

#### Half-pinch + Half-2p injury assay

Larvae were pinched according to the details listed above in “Pinch Injury Assay.” One to two hours later, larvae were mounted as detailed above and the pinch injured neuron was subjected to 2p laser injury. By 2 h after pinch injury, the blebbing, degradation, and removal of damaged branches were observed before reliably performing a secondary 2p laser injury. All remaining branches were removed. After laser injury, individual larvae were housed in grape plates with yeast paste and maintained at 20°C and 70% humidity.

#### Imaging

Larvae were immobilized for mounting by being sandwiched between an agarose pad and coverslip held together with vacuum grease (Thompson-Peer et al., 2016; Duarte et al., 2024; Prange et al., 2024; Hwu et al., 2025). Glycerol was used as the mounting media. Images of injured and within-animal uninjured control neurons were taken on a Zeiss LSM700 confocal microscope, Zeiss LSM780 confocal microscope, Zeiss LSM900 confocal microscope, or a Zeiss LSM980 confocal microscope. For Extended Data Figures 5-1 and 5-2 only, animals were imaged 1–2 h before injury. Neurons were imaged at 24 h AI to confirm injury. Neurons were imaged at 72 h AI to observe the end time point of regeneration. Only neurons with obvious survival and absence of half the dendritic arbor were included in subsequent analyses and presented data.

### Quantification and statistical analysis

Dendrite arbors were traced using the Simple Neurite Tracer (SNT) plugin in ImageJ to determine the number of dendrite branch tips and the total length of all the dendrite branches (Schindelin et al., 2012). The polygon selection tool in ImageJ was used to select and measure the total area of the dendritic arbor. The multipoint tool in ImageJ was used to count and label hemocytes. For UI control neurons, hemocytes were only included for quantification if they were located under the uninjured arbor. For pinched neurons, hemocytes were only included for quantification if they were located under the “spared” side of the arbor or were present in the area that the arbor would have covered, as if the neuron had been uninjured. Hemocyte quantification was split between “spared” and “injured” sides for pinched neurons and between two sections of “one-half total arbor” for UI controls. These values were determined by dividing the arbors in half, using the axon as a midpoint guideline.

For all graphs comparing means, values are plotted as mean ± standard error of the mean (SEM). For all graphs comparing means, individual data points represent individual neurons, except for Extended Data Figure 1-1*A*, in which individual data points represent the average time spent performing the pinch or half-2p injury assay. For paired data in Figure 1*E,G*, values are plotted for individual neurons with faded lines connecting the within-animal uninjured control neurons to each pinched neuron at 24 h AI. Larger, darker-colored points at 24 and 72 h AI represent the mean value. For paired data in Extended Data Figure 4-3*B*, *D*, and *F*, values are plotted for individual neurons with lines connecting repeated measurements of the same neuron at 24 and 72 h AI. For paired data in Figure 2*B*,*C*,*F*,*G* and Extended Data Figures 3-1*B*, 3-1*C*, 4-6*B*, and 4-6*D*, values are plotted for individual neurons with faded lines connecting repeated measurements of the same neuron at 24 and 72 h AI. Larger, darker-colored points at 24 and 72 h AI represent the mean value. Solid lines in Figure 2*B*,*C*,*F*,*G* and Extended Data Figure 3-1*B*,*C* represent the slope (*m*) between the mean 24 and 72 h AI values, which was calculated using the following equation:

m=(meanvalueat72hAI−meanvalueat24hAI)/48h.

Fold change in branch number was calculated using the following equation:

Foldchange=#ofbranchesat72hAI/#ofbranchesat24hAI.

To calculate area coverage, we used the polygon selection tool in Fiji and used the axon as a midpoint guideline for which branches to include as part of the injured half of the arbor. Fold change in area coverage was calculated using the following equation:

Foldchange=areaat72hAI/areaat24hAI.

To count the epidermal cells, we counted six apodemes on either side of the hemisegment, centered around the neuron's cell body, then drew “lines” between the two top apodemes and the two bottom apodemes. We counted all epidermal cells within the area created.

Normalized *F* (fluorescence) of A58-driven vkg-GFP was calculated using the following equation:

NormalizedF=Fgrayvalue/meanbackgroundF.

If class IV ddaC or class I ddaE neurons expressing CaMPARI experienced photobleaching due to capturing multiple images, they were removed from the dataset. If multiple images had to be captured, the first image captured was used for analysis. Normalized *F* (fluorescence) of red converted CaMPARI was calculated using the following equation:

NormalizedFofConvertedCaMPARI=Fmeanvalue/meanbackgroundF.

Δ*F*/*F*_background_ was calculated using the following equation:

ΔF/Fbackground=(medianFat72hrsAI−medianFat24hrsAI)/medianbackgroundF.

Sample sizes for all figures are as follows. Figure 1*E* uninjured control *n* = 4, pinch *n* = 4; Figure 1*G* uninjured control *n* = 10, pinch *n* = 10; Extended Data Figure 1-1*A* 2p *n* = 3, pinch *n* = 3; Figure 2*B* *n* = 10; Figure 2*C* *n* = 11; Figure 2*D* uninjured control *n* = 10, pinch *n* = 11; Figure 2*F* *n* = 14; Figure 2*G* *n* = 16; Figure 2*H* uninjured control *n* = 14, pinch *n* = 16; Figure 3*C* pinch *n* = 11, 2p *n* = 13; Figure 3*D* pinch *n* = 11, 2p *n* = 13; Figure 3*G* *n* = 9; Figure 3*H* *n* = 9; Extended Data Figure 3-1*B* *n* = 12; Extended Data Figure 3-1*C* *n* = 13; Extended Data Figure 3-1*D* uninjured control *n* = 12, 2p *n* = 13; Figure 4*B* 2p *n* = 8, pinch *n* = 3; Figure 4*C* 2p *n* = 6, pinch *n* = 4; Figure 4*D* 2p at 24 h AI *n* = 6, 2p at 72 h AI *n* = 6, pinch at 24 h AI *n* = 7, pinch at 72 h AI *n* = 4; Figure 4*E* 2p *n* = 4, pinch *n* = 4; Figure 4*F* uninjured control *n* = 5, 2p *n* = 4, pinch *n* = 4; Figure 4*G* 2p *n* = 3, pinch *n* = 2; Figure 4*H* 2p *n* = 5, pinch *n* = 7; Extended Data Figure 4-1*A* *n* = 7; Extended Data Figure 4-1*B* *n* = 3; Extended Data Figure 4-1*C* *n* = 8; Extended Data Figure 4-2*A* and *B* uninjured control at 24 h AI *n* = 8, uninjured control at 72 h AI *n* = 6; Extended Data Figure 4-2*C* and *D* pinch at 24 h AI *n* = 7, pinch at 72 h AI *n* = 4; Extended Data Figure 4-2*E* uninjured control at 24 h AI *n* = 5, pinch at 24 h AI *n* = 7, uninjured control at 72 h AI *n* = 3, pinch at 72 h AI *n* = 4; Extended Data Figure 4-2*F* 2p at 24 h AI *n* = 6, 2p at 72 h AI *n* = 6; Extended Data Figure 4-2*G* uninjured control at 24 h AI *n* = 3, 2p at 24 h AI *n* = 6, uninjured control at 72 h AI *n* = 3, 2p at 72 h AI *n* = 6; Extended Data Figure 4-3*A* *n* = 5; Extended Data Figure 4-3*B* *n* = 5; Extended Data Figure 4-3*C* *n* = 4; Extended Data Figure 4-3*D* *n* = 4; Extended Data Figure 4-3*E* *n* = 4; Extended Data Figure 4-3*F* *n* = 4; Extended Data Figure 4-3*G* uninjured control *n* = 1, pinch *n* = 1; Extended Data Figure 4-4*A* *n* = 5; Extended Data Figure 4-4*B* *n* = 2; Extended Data Figure 4-4*C* *n* = 3; Extended Data Figure 4-5*A* *n* = 11; Extended Data Figure 4-5*B* *n* = 7; Extended Data Figure 4-5*C* *n* = 5; Extended Data Figure 4-6*A* and *B* *n* = 8; Extended Data Figure 4-6*C* and *D* *n* = 8; Extended Data Figure 4-6*E* uninjured control at 24 h AI *n* = 8, pinch at 24 h AI *n* = 8, uninjured control at 72 h AI *n* = 8, pinch at 72 h AI *n* = 8; Figure 5*A* and *B* *n* = 4; Figure 5*C* and *D* *n* = 3; Extended Data Figure 5-1*A* *n* = 4; Extended Data Figure 5-1*B* *n* = 4; Extended Data Figure 5-2*A* *n* = 1; Extended Data Figure 5-2*B* *n* = 4.

All sample sizes for paired graphs of 24 to 72 h have the same *n* and are analyzing the same neurons at both time points, except for the datasets collected for the A58-driven ECM (a few of which are missing the 72 h after injury time point). Nearly all injured neurons have a within-animal uninjured control neuron.

If necessary, image processing using the freehand selection tool in Fiji was performed to remove denticle belts in order to better visualize the dendrites beneath/near those structures. All images displayed are representative of the phenotype. Representative images were selected for their clarity.

Some schematics were created with BioRender.

Statistical analysis was performed using GraphPad Prism software (version 10.3.1). An unpaired Student's *t* test was used for analyses comparing two groups when both groups passed a D'Agostino–Pearson omnibus normality test. A two-tailed Welch's Student's *t* test was used for all analyses comparing two groups when one or both groups did not pass a D'Agostino–Pearson omnibus normality test. A paired Student's *t* test was used for all analysis comparing the same neuron at two time points. A one-way ANOVA with Holm–Šidák's multiple-comparisons correction was used for analyses comparing more than two groups, and a two-way ANOVA with Holm–Šidák's multiple-comparisons correction was used for analyses with more than two variables. For all statistical tests, *p < 0.05, **p < 0.01, ***p < 0.001, ****p < 0.0001, and ^ns^*p* > 0.05.

Statistical tests for all figures are as follows. Details of each test are included in Extended Data Table 1-1. Figure 1*E*: paired *t* test; Figure 1*G*: paired *t* test; Extended Data Figure 1-1*A*: unpaired *t* test; Figure 2*B*: number of branch tips = paired *t* test, total branch length = paired *t* test; Figure 2*C*: number of branch tips = paired *t* test, total branch length = paired *t* test; Figure 2*D*: unpaired *t* test; Figure 2*F*: number of branch tips = paired *t* test, total branch length = paired *t* test; Figure 2*G*: number of branch tips = paired *t* test, total branch length = paired *t* test; Figure 2*H*: unpaired *t* test with Welch's correction; Figure 3*C*: number of branch tips = ordinary one-way ANOVA with Holm–Šidák's multiple-comparisons test, total branch length = ordinary one-way ANOVA with Holm–Šidák's multiple-comparisons test; Figure 3*D*: unpaired *t* test; Figure 3*G*: number of branch tips = ordinary one-way ANOVA with Holm–Šidák's multiple-comparisons test, total branch length = ordinary one-way ANOVA with Holm–Šidák's multiple-comparisons test; Figure 3*H*: ordinary one-way ANOVA with Holm–Šidák's multiple-comparisons test; Extended Data Figure 3-1*B*: number of branch tips = paired *t* test, total branch length = paired *t* test; Extended Data Figure 3-1*C*: number of branch tips = paired *t* test, total branch length = paired *t* test; Extended Data Figure 3-1*D*: unpaired *t* test with Welch's correction; Figure 4*D*: two-way ANOVA with Holm–Šidák's multiple-comparisons test; Figure 4*F*: ordinary one-way ANOVA with Holm–Šidák's multiple-comparisons test; Extended Data Figure 4-2*E*: two-way ANOVA with Holm–Šidák's multiple-comparisons test; Extended Data Figure 4-2*G*: two-way RM ANOVA with Holm–Šidák's multiple-comparisons test; Extended Data Figure 4-3*B*: paired *t* test; Extended Data Figure 4-3*D*: paired *t* test. Extended Data Figure 4-3*F*: paired *t* test; Extended Data Figure 4-6*B*: one-half total arbor = paired *t* test, one-half total arbor = paired *t* test, total arbor = paired *t* test; Extended Data Figure 4-6*D*: spared side = paired *t* test, injured side = paired *t* test, spared and injured sides = paired *t* test; Extended Data Figure 4-6*E*: 24 h AI = two-way ANOVA with Holm–Šidák's multiple-comparisons test, 72 h AI = two-way ANOVA with Holm–Šidák's multiple-comparisons test; Figure 5*B*: ordinary one-way ANOVA with Holm–Šidák's multiple-comparisons test; Figure 5*D*: ordinary one-way ANOVA with Holm–Šidák's multiple-comparisons test.

### Data availability

The datasets generated and/or analyzed during the current study are available from the lead contact upon reasonable request. Traces of dendrite arbors will be uploaded to Neuromorpho.org and available there. This paper does not report original code. Any additional information required to reanalyze the data reported in this paper is available from the lead contact upon request.

## Results

### Pinch injury damages da PNS neurons in *D. melanogaster* larvae

To develop an injury model that better mimics widespread tissue damage, we adapted a pinch injury method established by Burra et al. (2013). We modified the injury method such that cuticular puncture did not occur (Fig. 1*A*). To do this, we blunted a pair of #5 forceps and pinched the tissue laterally so that the larva's cuticle and epithelia bulged out between the tines of the forceps, which we termed a “larval muffin top” (Fig. 1*A*,*B*). The pinch injury was held for 10 s and typically lifted the larvae up into the air (Fig. 1*B*; Movies 1, 2). Compared with the traditional dendrite severing by a 2-photon (2p) laser, our new pinch injury method was significantly faster to perform (Extended Data Fig. 1-1*A*; 17.2 min per injury for 2p vs 4.2 min per injury for pinch). Throughout this manuscript, we will compare pinch-injured neurons to uninjured neurons and/or 2p-injured neurons. As such, representative images and data from pinch-injured neurons will be presented in green, uninjured neurons will be presented in purple, and 2p-injured neurons will be presented in blue. All images of neurons will be oriented such that the axon is pointing down and the region damaged by pinch will always be on the right side of the neuron/image.

**Figure 1.**
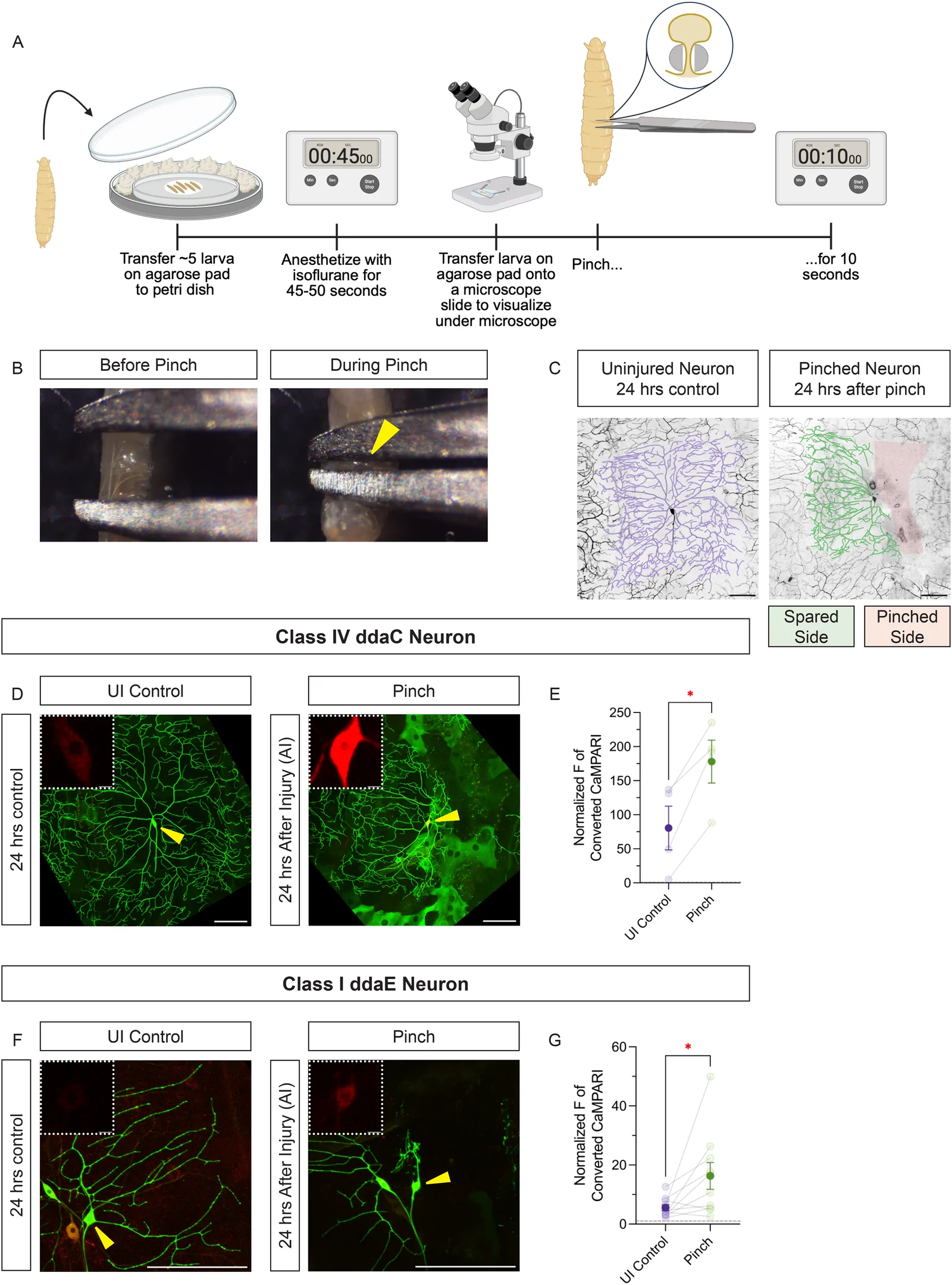
Pinch injury damages peripheral sensory neurons in *Drosophila* larvae. ***A***, Schematic of pinch injury technique. ***B***, Larvae before and during pinch injury. Yellow arrowhead points to larval “muffin top” formed between tines of forceps. ***C***, An uninjured (UI) class IV ddaC neuron at 24 h control and a pinched injured neuron at 24 h after pinch injury. Pinch injury removes half of the dendritic arbor (red-shaded area), leaving the other half spared (remaining green branches). Scale bar, 100 µm. ***D***, Uninjured (UI) within-animal control neurons 24 h control and pinched neurons 24 h after injury (AI), expressing both CD4-tdGFP and CaMPARI, which photoconverts from green (unconverted CaMPARI) to red (converted CaMPARI). Yellow arrowhead points to soma of UI and pinched ddaC neurons. Scale bar, 100 µm for composite channels. Scale bar, 5 µm for inset of cell body in top left corner. ***E***, Quantification of only red photoconverted CaMPARI fluorescence (normalized to background fluorescence) in uninjured (UI) within-animal control neurons (purple) and pinched neurons (green) at 24 h control or AI. ***F***, Same as ***D***, but class I ddaE neurons. ***G***, Same as ***E***, but class I ddaE neurons. Data for timing data for both injury paradigms is detailed in Extended Data Figure 1-1. Data demonstrating reproducibility of the pinch injury across users is detailed in Extended Data Figure 1-2.

10.1523/ENEURO.0058-26.2026.f1-1 Figure 1-1 P**i**nch **Injury Technique Is Faster to Perform Than 2p Dendrite Severing.** **(A)** Minutes required to perform injury to 1 neuron per animal for 2-photon (2p) injury assay (blue) and pinch injury assay (green). Download Figure 1-1, TIF file.

10.1523/ENEURO.0058-26.2026.f1-2 Figure 1-2 **Pinch Injury Technique Is Reproducible Across Different Users.** **(A)** Examples of pinch injury at 24 hrs after injury (AI), performed by three different individuals. Images shown are all class IV ddaC neurons expressing only CD4-tdGFP. Scale bar 100 µm. Download Figure 1-2, TIF file.

Following pinch injury, we first examined the extent of damage sustained by the PNS dendritic arborization (da) sensory neurons. We visualized the class IV da neuron, ddaC, using cell type-specific expression of a membrane-tagged GFP and imaged them at 24 h after pinch injury. We found that abdominal segment ddaC neuron A4 or A5 was successfully injured by our approach: one lateral half of the dendritic arbor was damaged, and the other half was left intact (Fig. 1*C*). Typically, we observed that two neurons were damaged by pinch injury. Most often, one neuron was successfully pinched, wherein half of the dendritic arbor had been removed, while the other neuron was damaged to a lesser extent, missing only part of the dendritic arbor. We did occasionally observe successful pinch injuries to neurons in both hemisegments A4 and A5, but this was not typical. Importantly, we found this pinch technique to be reproducible across different users (Extended Data Fig. 1-2*A*; examples of pinch injuries performed by three different persons). Figure 1*C* identifies the pinch-injured region (red-shaded area).

Next, we set out to determine whether pinch injury led to calcium influx, the first step of dendrite injury detection in *da* neurons, as we and others have shown previously (Duarte et al., 2024; Hertzler et al., 2024). We used CaMPARI (Calcium-Modulated Photoactivatable Ratiometric Indicator), a photoactivatable calcium indicator that photoconverts from green to red only in the simultaneous presence of calcium ions and UV light (Fosque et al., 2015). Immediately following pinch injury, larvae expressing CaMPARI in ddaC neurons were exposed to 20 s of UV light. Red converted CaMPARI fluorescence was detected in the soma and proximal primary dendrites in pinched ddaC neurons, but not in uninjured (UI) control neurons (Fig. 1*D*; Extended Data Table 1-1 details each statistical test performed and should be referred to for all figures presented in this manuscript). When we quantified the increase in red photoconverted CaMPARI fluorescence, pinched ddaC neurons had a significantly higher red fluorescence intensity of converted CaMPARI compared with uninjured controls (Fig. 1*E*). Class IV ddaC neurons function as nociceptors, involving a transient influx of Ca^2+^ into the dendrite as a response to pain and/or harsh touch (Hwang et al., 2007; Terada et al., 2016). To determine if the calcium influx detected by CaMPARI is due to injury or nociception, we repeated these pinch injuries with CaMPARI in the non-nociceptive class I ddaE proprioceptive neurons (Hughes and Thomas, 2007; Song et al., 2007; Cheng et al., 2010; He et al., 2019). Red converted CaMPARI fluorescence was detected in the soma of pinched class I ddaE neurons, but not uninjured control neurons (Fig. 1*F*). Quantification of the increase in red photoconverted CaMPARI fluorescence demonstrated that pinched ddaE neurons had a significantly higher red fluorescence intensity of converted CaMPARI compared with UI controls (Fig. 1*G*). These results demonstrate that Ca^2+^ influx due to pinch injury occurs in both proprioceptive and nociceptive neurons, strongly supporting the interpretation that Ca^2+^ influx is a response to dendrite injury rather than to nociceptive sensory activation. We conclude that the pinch technique causes calcium influx due to dendrite injury in these neurons, which is a critical step in dendrite injury detection and is similar to what has been observed immediately following 2p injury (Duarte et al., 2024; Hertzler et al., 2024).

Together, these results demonstrate that pinching injury can damage dendrites, in a highly reproducible and rapid manner.

### ddaC and ddaE neurons (re)grow their branches following pinch injury

To assess how da neuronal dendrites respond to pinch injury, we first decided to evaluate the dendritic arbors of the class IV ddaC neurons. The dendritic arbors of class IV ddaC neurons are large, filling their receptive field by 48 h after egg lay (AEL) and are very dynamic (Grueber et al., 2002; Parrish et al., 2009). Further, they retain a robust capacity for dendrite regeneration (Song et al., 2012; Stone et al., 2014; Thompson-Peer et al., 2016; Hertzler et al., 2023; Duarte et al., 2024; Prange et al., 2024; Hwu et al., 2025). Pinch injury damaged half of the dendrite arbor and left the other half uninjured (Fig. 2*A*). Following pinch injury, the spared half of the arbor became visibly bushier as it gained branches over time (Fig. 2*A*). Specifically, ddaC neurons increased in total branch number and total dendrite length from 24 to 72 h after injury (AI; Fig. 2*A*,*C*). The growth on the spared half of the arbor was roughly similar to uninjured ddaC neurons (Fig. 2*A*,*B*), which continue to gain branches over time as the larvae grow and develop (Parrish et al., 2009).

**Figure 2.**
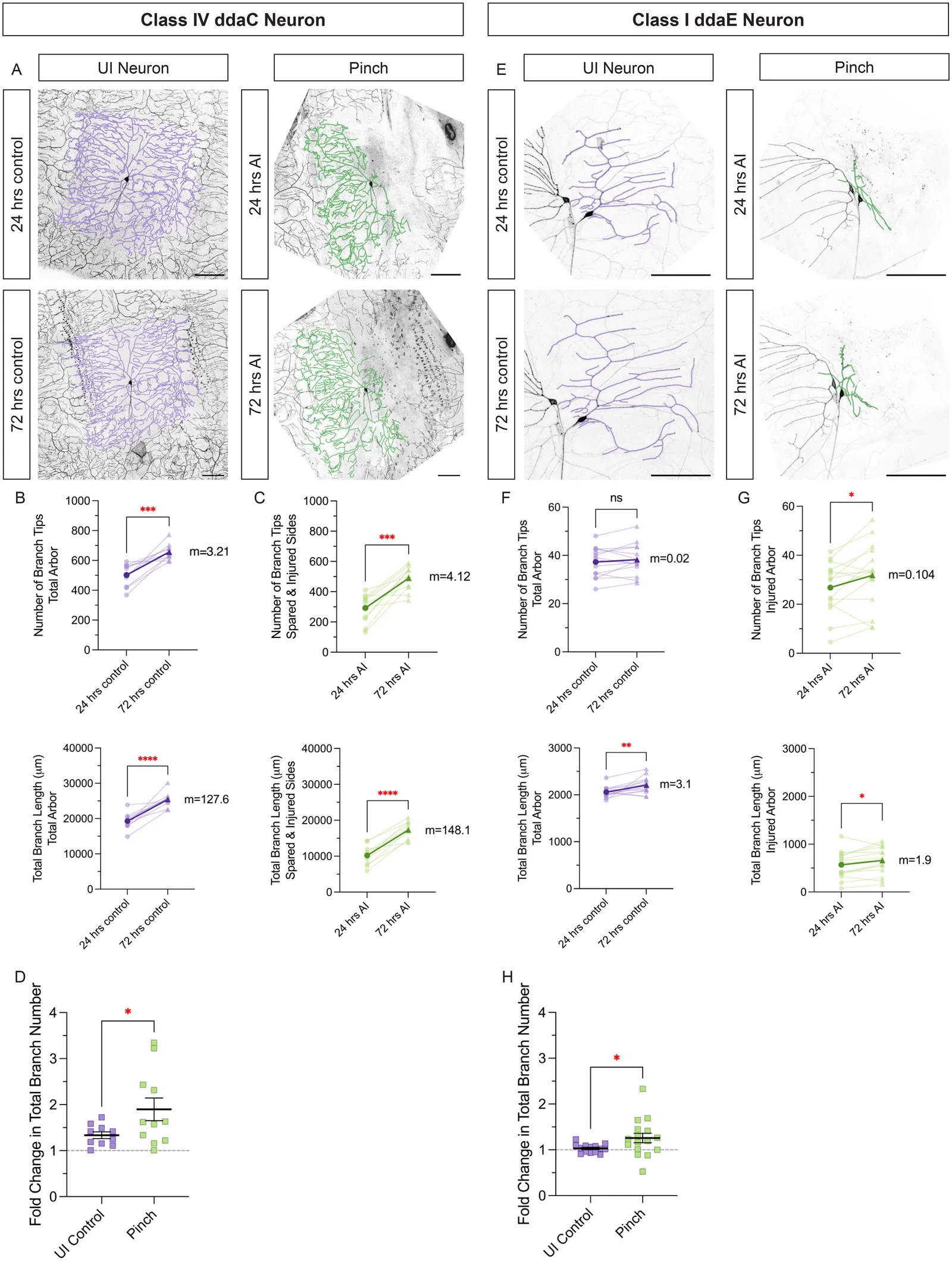
ddaC and ddaE neurons grow following pinch injury. ***A***, Class IV ddaC uninjured (UI) within-animal control (purple) and pinched (green) neurons at 24 and 72 h control or AI. Scale bar, 100 µm. ***B***, ***C***, Number of branch tips (top) and total branch length (bottom) of UI neurons (***B***) and pinch injured class IV ddaC neurons (***C***) at 24 and 72 h control or AI. ***D***, Fold change in total branch number of UI and pinched class IV ddaC neurons. ***E***, Same as ***A***, but class I ddaE neurons. Scale bar, 100 µm. ***F***, ***G***, Same as ***B***, ***C***, but class I ddaE neurons. ***H***, Same as ***D***, but class I ddaE neurons.

In order to determine if the branches gained on the spared half of the pinched neuron were stimulated by injury or were simply the branch growth of normal development, we took two approaches. First, we looked at class I ddaE neurons, which are neurons that establish their arbors by 24 h AEL and are very static during later development (Sugimura et al., 2003). In the absence of injury, they do not change in branch number over time, though they do increase in branch length as the larvae grow (Fig. 2*E*,*F*). Thus, any significant increase in branch number following pinch injury would be indicative of regeneration. We found that pinched class I ddaE neurons did increase in total branch number and total dendrite length from 24 to 72 h after pinch, compared with the stable uninjured class I ddaE neurons that did not increase in total branch number during this time (Fig. 2*E*,*F*,*G*). Because asymmetric ddaE neurons lack a spared side, the injured arbor is the total dendrite arbor. As a second approach, we evaluated the growth rate of each neuron subtype, both class IV ddaC and class I ddaE neurons. We found that both had a greater fold change in total branch number (total branch number at 72 h AI divided by total branch number at 24 h AI) following pinch injury compared with their uninjured controls (Fig. 2*D*,*H*).

This result coupled with the regenerative response of class I ddaE neurons following pinch injury is indicative that both class IV ddaC and class I ddaE neurons exhibit regeneration of their dendrites following pinch injury and not simply continued developmental growth.

### ddaC neurons preferentially regenerate into undamaged tissue

Next, we wanted to compare regeneration following pinch injury versus 2p injury, as dendrite severing by a high-powered laser is the “gold standard” for precise injury in the field (Hertzler and Rolls, 2024). To compare the two injury methods, we mimicked the amount of dendrite removal obtained with a pinch injury by using a 2p laser to cut off half of the class IV dendritic arbor (Fig. 3*A*,*B*; Extended Data Fig. 3-1*A*). We called this injury a “half-2p bald” as half of the neuron was left “bald” by removal of half of its dendrite arbor. Class IV ddaC neurons regenerated following this half-2p bald laser injury. They increased their total branch number and total branch length, and they had a significantly higher fold change in total branch number compared with their UI controls (Fig. 3*B*; Extended Data Figs. 3-1*B*–3-1*D*).

**Figure 3.**
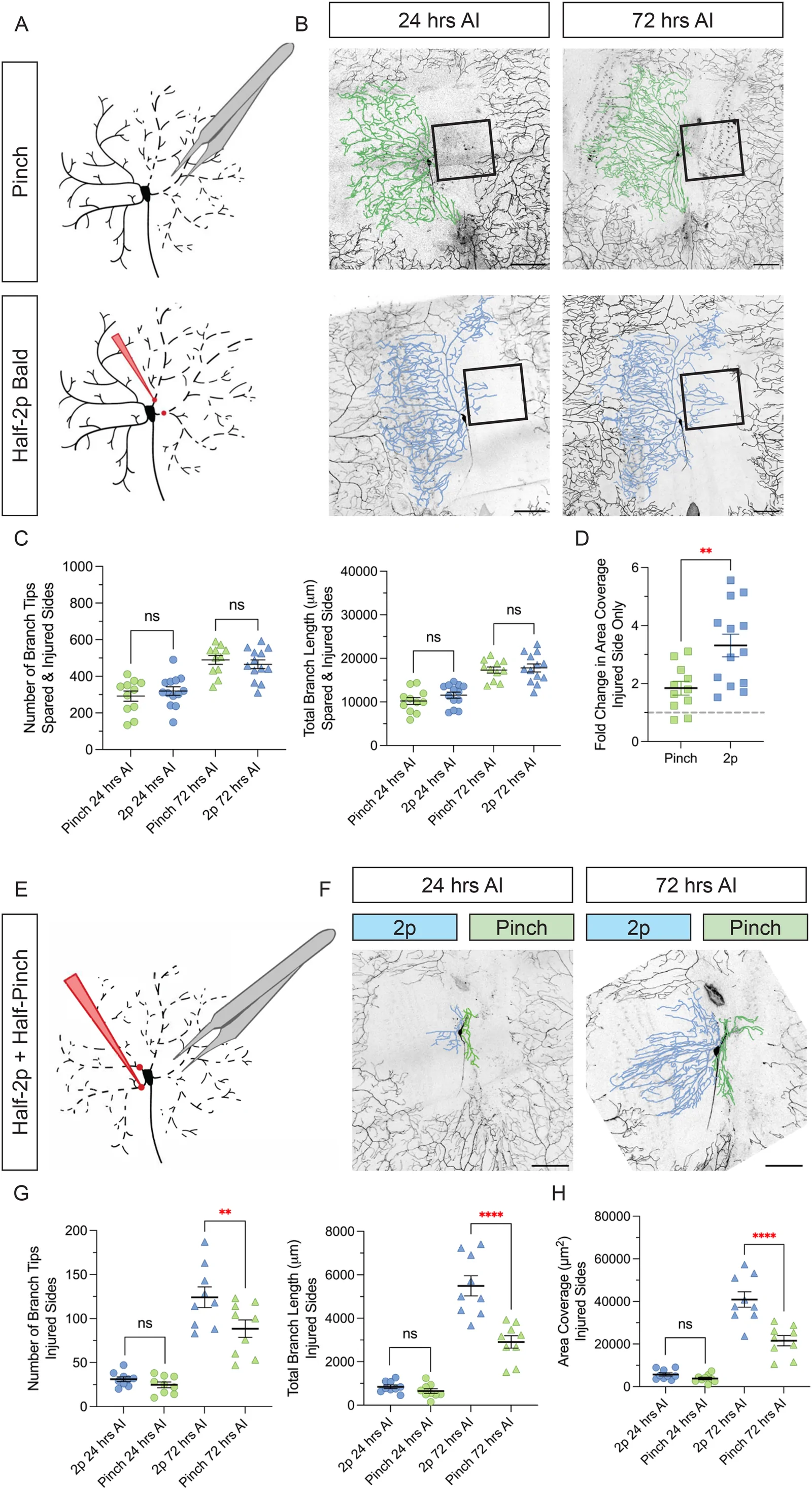
ddaC neurons preferentially regenerate into empty territory following 2p injury. ***A***, Schematics of pinch (top) and half-2p bald (bottom) injuries. ***B***, Pinch (top) and half-2p bald (bottom) injured class IV ddaC neurons at 24 and 72 h AI. Black boxes identify empty territory. Scale bar, 100 µm. ***C***, Number of branch tips (left) and total branch length (right) of class IV ddaC neurons injured by 2p (blue) or pinch (green). ***D***, Fold change in area coverage of the injured half only of individual neurons following 2p or pinch injury. ***E***, Schematic of a “half-pinch + half-2p” injury on the same class IV ddaC neuron. ***F***, Half-2p (left side) + half-pinch (right side) injured class IV ddaC neuron at 24 and 72 h AI. Scale bar, 100 µm. ***G***, Number of branch tips (left) and total branch length (right) of 2p injured side (blue) and pinch injured side (green) of individual class IV ddaC neurons at 24 and 72 h AI that were injured by both injury paradigms. ***H***, Area coverage (µm^2^) of 2p injured side (blue) and pinch injured side (green) of individual class IV ddaC neurons at 24 and 72 h AI that were injured by both injury paradigms. Regeneration data following half-2p bald is shown in Extended Data Figure 3-1.

10.1523/ENEURO.0058-26.2026.f3-1 Figure 3-1 **Half-2p Bald class IV ddaC Neurons Grow Into Undamaged Tissue.** **(A)** Class IV ddaC uninjured (UI) within-animal control (purple) and half-2p (blue) injured neurons at 24 and 72 hrs control or AI. Scale bar 100 µm. **(B-C)** Number of branch tips (top) and total branch length (bottom) of uninjured (UI) within-animal control (B, purple) and half-2p bald (C, blue) neurons 24 to 72 hrs control or AI. **(D)** Fold change in total branch number of uninjured (UI) within-animal control (purple) and half-2p bald (blue) class IV ddaC neurons. Download Figure 3-1, TIF file.

When comparing the total number of branches and total dendrite length between pinched and half-2p bald class IV ddaC neurons, there were no significant differences at 24 or 72 h AI in the total number or length of dendrites (Fig. 3*C*). We noticed, however, that pinch injury resulted in fewer dendrites innervating the empty territory on the injured side relative to half-2p bald neurons (Fig. 3*B*, black boxes). Since this phenotype was not captured by calculating the total number of branches or total dendrite length (Fig. 3*C*), we therefore calculated the fold change in area coverage of only the injured side of the arbor. Neurons injured by half-2p bald experienced a greater fold change in area coverage on their injured side, reflecting our observation of an increased capacity for innervating empty territory following injury (Fig. 3*D*).

Given this difference in regenerative capacity, we next wanted to challenge individual neurons to regenerate their dendrites following both pinch and 2p injury. Class IV ddaC neurons were first pinched and 1–2 h later, the remaining arbor (the other half) was cut off with the 2p laser. This specific “half-pinch + half-2p” injury paradigm removed all branches from the class IV ddaC neuron and forced new dendrites to choose to regrow on one side or the other (Figs. 3*E*,*F*). For all quantifications of this injury paradigm, the axon was used as a midpoint guideline to separate the two halves of the arbor. Following this half-pinch + half-2p injury, there was no statistical difference between the two sides in branch number, dendrite length, or area coverage at 24 h AI (Fig. 3*G*,*H*). However, at 72 h AI, the half of the arbor responding to 2p injury regenerated significantly more than the half of the arbor responding to pinch injury, with respect to branch number, dendrite length, and area coverage (Fig. 3*G*,*H*).

Taking the results of these two experiments together, we conclude that ddaC neurons preferentially regenerate into the empty territory that had been injured by 2p injury. Since challenging individual neurons to regenerate following both injury paradigms led to differential regenerative outcomes, we hypothesized that extrinsic mechanisms may be causing poor reinnervation following pinch injury, either by active inhibition or absence of support.

### Pinch injury damages other cell types and surrounding tissues

Following the differential regeneration of dendrites observed from the half-pinch + half-2p experiments, we next evaluated how the extracellular environment might be affecting regeneration following both injury paradigms. We examined the effects of 2p and half-pinch injury individually on the surrounding tissues (Fig. 4) and then examined tissue damage after combined half-pinch + half-2p injury together (Fig. 5). There are a number of cell types and tissues that interact with the da neurons (Fig. 4*A*). The da neuronal dendrites are suspended in an extracellular matrix (ECM) and innervate the larval epidermis. Glia wrap the axon, cell body, and proximal dendrites of the da neurons, and resident immune cells, hemocytes, are present. First, we examined the ECM.

**Figure 4.**
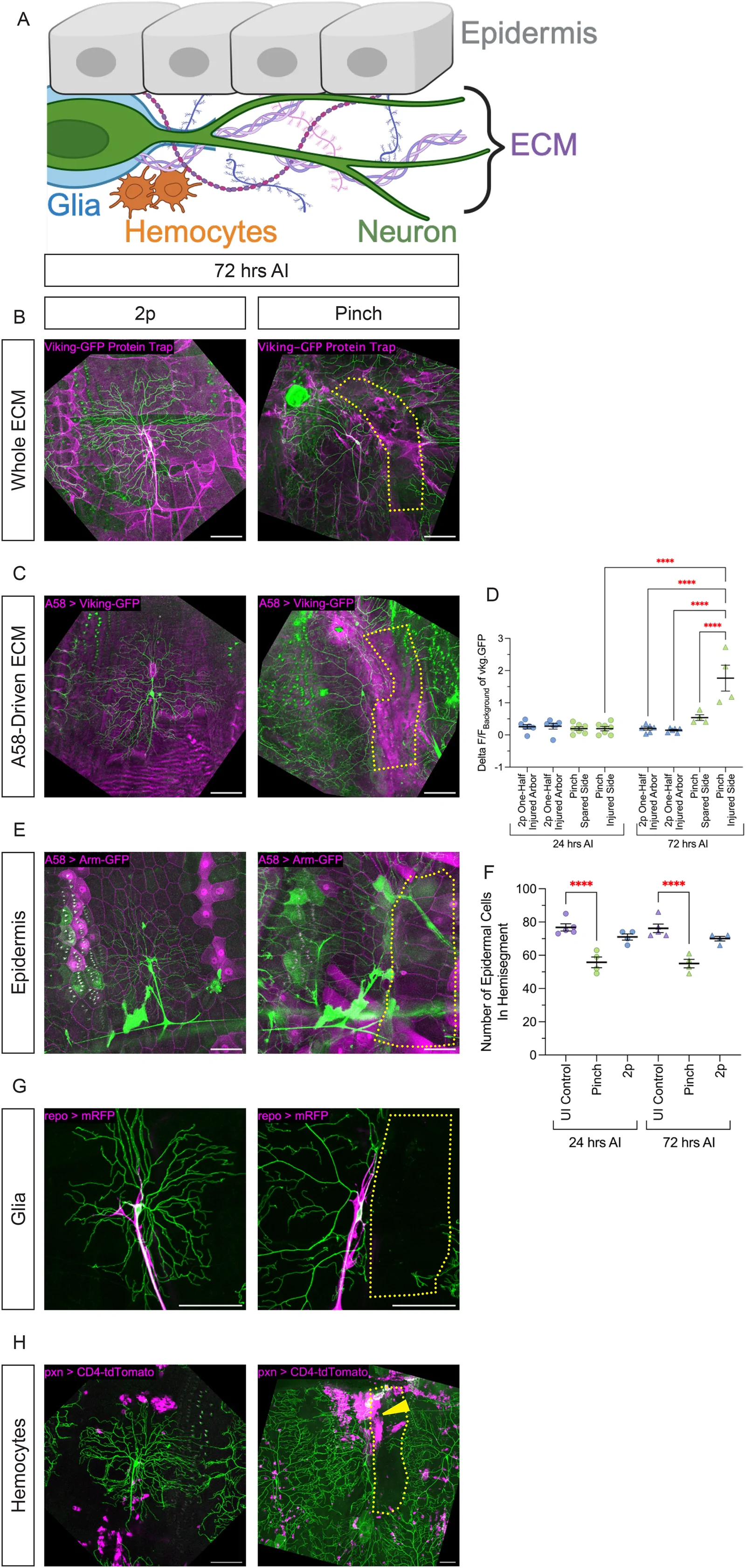
Pinch injury damages ECM, epidermal cells, and recruits hemocytes, but does not appear to damage glia. ***A***, Schematic of a class IV ddaC neuron (green) with the other cell types and tissues it interacts with: epidermis (gray), glia (blue), ECM (purple), and hemocytes (orange). ***B***, ***C***, ***E***, ***G***, ***H***, ddaC neurons (green) with other tissues in magenta following 2p-full bald (left) or pinch (right) injuries at 72 h AI. Dotted yellow outline highlights the region lacking dendrites due to pinch injury. ***B***, Whole ECM protein trap (vkg-GFP); see Extended Data Figure 4-1 for individual channels. ***C***, A58-driven ECM (Gal4^A58^ > vkg-GFP); see Extended Data Figure 4-2 for individual channels and quantification. ***E***, Epidermal tight junction marker (Gal4^A58^ > Armadillo-GFP); see Extended Data Figure 4-3 for individual channels and counts of epidermal cells. ***G***, Glial marker (Gal4^repo^ > CD4-tdTomato); see Extended Data Figure 4-4 for individual channels. ***H***, hemocyte marker (Gal4^pxn^ > CD4-tdTomato); see Extended Data Figure 4-5 for individual channels. Yellow arrowhead identifies hemocyte accumulation. Scale bar, 100 µm. ***D***, Δ*F*/*F*_background_ of vkg-GFP median fluorescent values following pinch (green) or 2p-full bald (blue) injuries at 24 and 72 h AI. Fluorescent values of pinched neurons are separated by pinch injured and spared sides. Fluorescent values of 2p-full bald neurons are arbitrarily separated into two sides, each representing “one-half injured arbor.” ***F***, Number of epidermal cells near uninjured (purple), pinched (green), or 2p-full bald (blue) neurons at 24 and 72 h AI. Individual channels of whole ECM images are shown in Extended Data Figure 4-1. Individual channels of A58-driven ECM images and quantification are shown in Extended Data Figure 4-2. Individual channels of epidermal cell images and counts are shown in Extended Data Figure 4-3. Individual channels of glial images are shown in Extended Data Figure 4-4. Individual channels of hemocyte images are shown in Extended Data Figure 4-5. Quantification of hemocyte data is shown in Extended Data Figure 4-6.

10.1523/ENEURO.0058-26.2026.f4-1 Figure 4-1 **Pinch Injury, but Not 2p-Full Bald Injury, Damages Whole ECM (viking/Collagen IV).** **(A-C)** Uninjured (A), pinched (B), or 2p-full bald (C) class IV ddaC neurons with an ECM Protein Trap (vkg-GFP) at 24 and 72 hrs control or AI. Dotted yellow outlines highlight the region lacking dendrites due to pinch injury in (B). Scale bar 100 µm. Composite images of pinched or 2p-full bald neurons at 72 hrs AI are also shown in Fig 4B. Download Figure 4-1, TIF file.

10.1523/ENEURO.0058-26.2026.f4-2 Figure 4-2 **Pinch Injury, but Not 2p-Full Bald Injury, Damages A58-Driven ECM (viking/Collagen IV).** **(A, C, F)** Uninjured (A), pinched (C), or 2p-full bald (F) class IV ddaC neurons with A58-driven ECM marker (Gal4^A58^ > vkg-GFP) at 24 and 72 hrs control or AI. Dotted yellow outlines highlight the region lacking dendrites due to pinch injury in (C). Composite images of pinched or 2p-full bald neurons at 72 hrs AI are also shown in Fig 4C. Scale bar 100 µm. **(B, D)** Normalized F of vkg-GFP over 250 µm starting from the cell body at 24 hrs control or AI (top) and 72 hrs control or AI (bottom). UI neurons (B) were arbitrarily separated into two sides, each representing “one-half total arbor”. Pinched neurons (D) were separated by spared (light green) and pinched (dark green) sides. **(E, G)** ΔF/F_background_ of vkg-GFP median fluorescence values at 24 and 72 hrs control or AI in uninjured (purple) or pinched (green) neurons (E), or uninjured (purple) and 2p-full bald (blue) neurons (G). Download Figure 4-2, TIF file.

10.1523/ENEURO.0058-26.2026.f4-3 Figure 4-3 **Pinch Injury, but Not 2p-Full Bald Injury, Disrupts Epidermal Cell Morphology.** **(A, C, E)** Uninjured (A), pinched (C), or 2p-full bald (E) class IV ddaC neurons with epidermal marker (Gal4^A58^ > UAS-Armadillo-GFP) at 24 and 72 hrs control or AI. Epidermal cells included for quantification are outlined in yellow. Composite images of pinched or 2p-full bald neurons at 72 hrs AI are also shown in Fig 4E. Scale bar 100 µm. **(B, D, F)** Number of epidermal cells in hemisegment at 24 and 72 hrs control or AI near uninjured (B), pinched (D), or 2p-full bald (F) neurons. Comparison among uninjured and injured neurons and at each time point is also shown in Fig 4F. **(G)** Brightfield images of the cuticle above an uninjured neuron at 72 hrs control or pinched neuron at 72 hrs AI. Download Figure 4-3, TIF file.

10.1523/ENEURO.0058-26.2026.f4-4 Figure 4-4 **Neither Pinch Nor 2p-Full Bald Injuries Seem to Dramatically Damage Glia.** **(A-C)** Uninjured (A), pinched (B), or 2p-full bald (C) class IV ddaC neurons with glial marker (Gal4^repo^ > mRFP) at 24 and 72 hrs control or AI. Dotted yellow outlines highlight the region lacking dendrites due to pinch injury in (B). Scale bar 100 µm. Composite images of pinched or 2p-full bald neurons at 72 hrs AI are also shown in Fig 4G. Download Figure 4-4, TIF file.

10.1523/ENEURO.0058-26.2026.f4-5 Figure 4-5 **Pinch Injury, but Not 2p-Full Bald Injury, Recruits Hemocytes.** **(A-C)** Uninjured (A), pinched (B), or 2p-full bald (C) class IV ddaC neurons with hemocyte marker (Gal4^pxn^ > CD4-tdTomato) at 24 and 72 hrs control or AI. In the hemocyte channel for the uninjured neurons at 24 and 72 hrs control and for the 2p-injured neurons at 24 and 72 hrs AI, the top yellow box identifies the dorsal vessel cluster, the middle yellow box identifies the dorsal stripe, and the bottom yellow box identifies the lateral patch of hemocytes. For pinch injured neurons at 24 and 72 hrs AI, yellow arrowheads identify hemocyte accumulation. Dotted yellow outlines highlight the region lacking dendrites due to pinch injury in (B). Scale bar 100 µm. Composite images of pinched or 2p-full bald neurons at 72 hrs AI are also shown in Fig 4H. Download Figure 4-5, TIF file.

10.1523/ENEURO.0058-26.2026.f4-6 Figure 4-6 **Pinch Injury Induces a Robust and Prolonged Immune Response.** **(A, C)** Uninjured (A) or pinched (C) class IV ddaC neurons with hemocyte marker (Gal4^pxn^ > RedStinger) at 24 and 72 hrs control or AI. In the hemocyte channel for the uninjured neurons at 72 hrs control, the top yellow box identifies the dorsal vessel cluster, the middle yellow box identifies the dorsal stripe, and the bottom yellow box identifies the lateral patch of hemocytes. Dotted yellow outlines highlight the region lacking dendrites due to pinch injury in (C). Scale bar 100 µm. **(B, D)** Number of hemocytes at 24 and 72 hrs control or AI within the two halves (left and middle) or total (right) region of the hemisegment of uninjured (B) or the injured side (left), spared side (middle), or total (right) region of the hemisegment of pinched (D) neurons. **(E)** Number of hemocytes within the hemisegment of uninjured (purple) or pinched (green) neurons at 24 (top) and 72 (bottom) hrs control or AI. Download Figure 4-6, TIF file.

**Figure 5.**
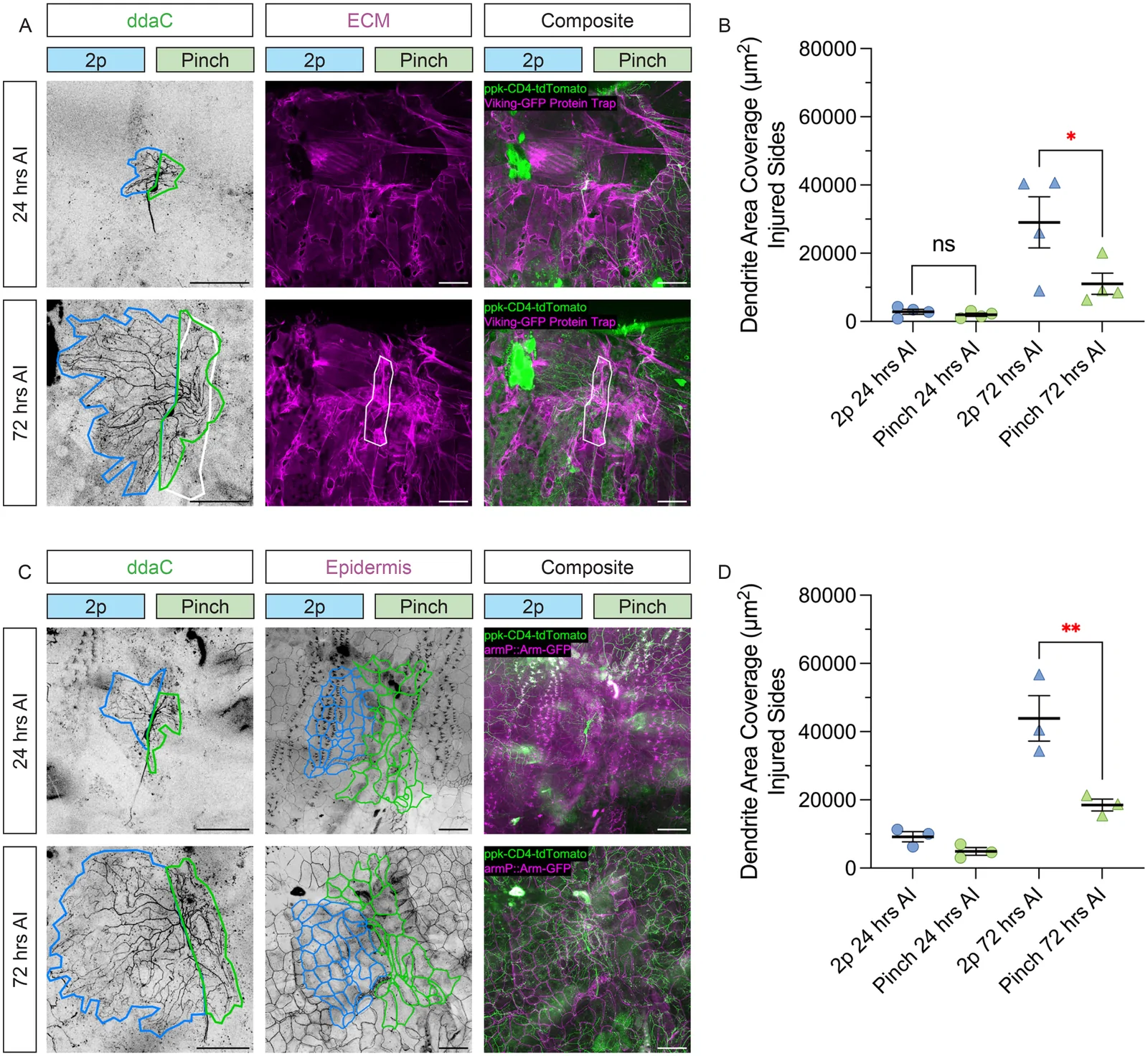
Surrounding tissue damage restricts dendrite regeneration. ***A***, ***C***, Half-2p (left side, blue) + half-pinch (right side, green) injured class IV ddaC neurons at 24 and 72 h AI with an ECM protein trap (vkg-GFP; ***A***) or epidermal marker (armP::Armadillo-GFP; ***C***). ddaC only images have been cropped for better visualization of neurons. Scar formation in the ECM at 72 h AI is outlined in white in ***A***. Blue (half-2p) and green (half-pinch) outlines are drawn around each injured half of the class IV ddaC neuron to represent quantified dendrite area coverage (µm^2^) in ***B*** and ***D***. Epidermal cells damaged by either injury paradigm (half-2p, blue; half-pinch, green) are outlined at both 24 and 72 h AI in ***C***. Scale bar, 100 µm. ***B***, ***D***, Dendrite area coverage (µm^2^) of 2p injured side (blue) and pinch injured side (green) individual class IV ddaC neurons injured by both injury paradigms at 24 and 72 h AI with an ECM Protein Trap (vkg-GFP; ***B***) or epidermal marker (armP::Armadillo-GFP; ***D***). Data highlighting epidermal cell morphology before and after pinch injury is shown in Extended Data Figure 5-1. Data demonstrating improved epidermal cell visualization with armP::Arm-GFP is shown in Extended Data Figure 5-2.

10.1523/ENEURO.0058-26.2026.f5-1 Figure 5-1 **Epidermal Cells Appear to Undergo Fusion Following Pinch Injury.** **(A, B)** Uninjured (A) or pinched (B) class IV ddaC neurons with epidermal marker (armP::Armadillo-GFP) before injury and 24 hrs control or AI. Epidermal cells are outlined in yellow. Dotted yellow outlines highlight the region lacking dendrites due to pinch injury in (B) at 24 hrs AI. Scale bar 100 µm. Download Figure 5-1, TIF file.

10.1523/ENEURO.0058-26.2026.f5-2 Figure 5-2 Arm-GFP Under Control of *arm* Promoter Labels Epidermal Cells Better Than UAS-controlled Arm-GFP Before and After Injury. **(A, B)** Class IV ddaC neurons with epidermal marker (Armadillo-GFP) under control of the Gal4-UAS system (A) or *arm* promoter (B) before injury and 24 hrs AI. Epidermal cells are outlined in yellow. Dotted yellow outlines highlight the region lacking dendrites due to pinch injury at 24 hrs AI. Scale bar 100 µm Download Figure 5-2, TIF file.

Throughout development, the larval da neurons adhere to components of the ECM through adhesion molecules like integrins (Kim et al., 2012). These interactions help constrain dendrites to a 2D space: within the ECM and between the musculature and epidermis (Grueber et al., 2002; Yasunaga et al., 2010; Han et al., 2012; Kim et al., 2012). Major components of the ECM include laminins and collagen IV, which are primarily produced by the fat body and hemocytes but also by the larval epidermis (Rodriguez et al., 1996; Han et al., 2012; Isabella and Horne-Badovinac, 2015; Ramos-Lewis et al., 2018).

Thus, to visualize the ECM, we chose to look at viking (vkg), which is homologous to the mammalian Collagen IV (Yasothornsrikul et al., 1997). We first used a protein trap fly line where the endogenous vkg gene is tagged with GFP. In uninjured hemisegments, vkg-GFP predominantly wrapped the muscle and muscle attachment sites; it also wrapped the axon, cell body, and proximal dendrites of the ddaC class IV neurons (Extended Data Fig. 4-1*A*). Following 2p injury, vkg-GFP was undamaged (Fig. 4*B*, Extended Data Fig. 4-1*C*). Following pinch injury, vkg-GFP was severely damaged (Fig. 4*B*, Extended Data Fig. 4-1*B*). The vkg-GFP wrapping the muscle and muscle attachment sites was significantly damaged, and there was no longer a consistent vkg-GFP signal across the hemisegment. Vkg-GFP continued to wrap the axon, cell body, and proximal dendrites of the spared half of the pinched arbor. However, there was a significant accumulation of vkg-GFP signal on the pinched half of the arbor that aligned with the pinched edge of the injured side of the arbor. This accumulation of vkg-GFP was reminiscent of a glial or fibrotic scar, which can create an inhibitory, nonpermissive environment for neurite regrowth (Liesi and Kauppila, 2002; Klapka and Müller, 2006; Tran et al., 2022).

Since the whole-body vkg-GFP had a significant amount of signal, mostly surrounding the muscle, it was difficult to see the ECM directly interacting with the dendrites. Thus, we sought to visualize vkg-GFP specifically expressed by A58-Gal4, which drives expression in the larval epidermis and, in later third instar stages, the fat body (Ramos-Lewis et al., 2018). Uninjured hemisegments had a smooth, even vkg-GFP fluorescence (*F*) signal that mimics the “cobblestone” pattern of the epidermis (Extended Data Fig. 4-2*A*, quantified in Extended Data Fig. 4-2*B*). The A58-driven vkg-GFP appeared unaltered by 2p injury (Fig. 4*C*, Extended Data Fig. 4-2*F*, quantified in Extended Data Fig. 4-2*G*). Thus, the A58-driven vkg-GFP was uninjured following 2p injury. When we compare pinched animals versus 2p injured animals (Fig. 4*D*) or versus uninjured animals (Extended Data Fig. 4-2*E*), we find that A58-driven vkg-GFP was enriched on the pinched side of the arbor compared with either half of an uninjured neuron or a neuron injured by 2p (Extended Data Fig. 4-2*C*). The enrichment of A58-driven vkg-GFP on the pinched half of the neuron increased from 24 to 72 h AI (Extended Data Fig. 4-2*D*,*E*). This was reminiscent of the enrichment of whole-body vkg-GFP observed in Figure 4*B*. As an additional note, we observed a slight increase in the A58-driven vkg-GFP fluorescence on the spared half at 72 h AI, most likely due to diffusion of vkg-GFP from the pinched side (Fig. 4*D*, Extended Data Fig. 4-2*D,E*). Thus, both endogenous and over-expressed viking accumulated and persisted at the site of injury, only after pinch injury.

Next, we wanted to look at the larval epidermis. The da neurons establish their arbors in a 2D space: within the ECM and between the musculature and epidermis (Grueber et al., 2002; Yasunaga et al., 2010; Han et al., 2012; Kim et al., 2012). The larval epidermis is a monolayer sheet of terminally differentiated epidermal cells and plays a crucial role in establishing the dendritic arbor of the da neurons (Parrish et al., 2009; Burra et al., 2013; Jiang et al., 2014; Poe et al., 2017). HSPGs expressed by the larval epidermis are required for stabilization of high-order dendrites in ddaC class IV neurons (Poe et al., 2017). Additionally, portions of ddaC class IV dendrite branches are enclosed by epidermal cells via Coracle to restrict dendrite outgrowth and mediate the occupancy of multiple classes of da neurons in a 2D space (Tenenbaum et al., 2017). To visualize the epidermis, we used a GFP-tagged version of Armadillo, the fly homolog of beta-catenin and an adherens-junction marker (Cox et al., 1996). Armadillo-GFP clearly outlined individual epidermal cells, and we additionally outlined epidermal cells included for quantification in yellow to highlight their morphology (Extended Data Figs. 4-3, 5-1). For uninjured hemisegments, the epidermis was a single sheet of polygonal epidermal cells (Extended Data Figs. 4-3*A*, 5-1*A*), and we found no change in the number of epidermal cells from 24 to 72 h control (Extended Data Fig. 4-3*B*). Following 2p injury, the epidermis appeared the same as uninjured hemisegments and did not change over time (Fig. 4*E*,*F*; Extended Data Fig. 4-3*E*,*F*). However, following pinch injury, the epidermis was damaged (Fig. 4*E*; Extended Data Figs. 4-3*C*, 5-1*B*, 5-2*B*). On the pinched side of the neuron, the epidermal cells were disfigured: close to the focal point of injury, they were small and crushed; next to the muscle attachment sites (apodemes), the epidermal cells were elongated and larger than normal. There were significantly fewer epidermal cells following pinch injury than there were for uninjured neurons (Fig. 4*F*). Even though we found damage to the epidermal cells following pinch injury, there was no damage to the overlying cuticle (Extended Data Fig. 4-3*G*). We examined the morphology of the epidermal cells before and after pinch injury (Extended Data Figs. 5-1, 5-2) and found that epidermal cells on the pinched side of the hemisegment could not be easily tracked or identified between images taken before injury (96 h AEL) and after injury (24 h AI; Extended Data Figs. 5-1*B*, 5-2*B*). On the spared side of a pinched hemisegment, the epidermal cells appeared uninjured, similar to uninjured and 2p-injured hemisegments, and did not change over time (Fig. 4*E*; Extended Data Figs. 4-3*D*, 5-1, 5-2*B*). Damage to epidermal cells may contribute to why pinch-injured neurons exhibit poor regrowth into empty territory.

Next, we looked at the glia. Dendritic arborization neurons have three layers of glia that wrap the axon, the soma, and the proximal region of the primary dendrites (Extended Data Fig. 4-4*A*; Yadav et al., 2019). Glia are important for establishing both the structure and function of ddaC class IV neurons. Following 2p injury, the glia were capable of wrapping the axon and soma (Thompson-Peer et al., 2016), but they seemed to have trouble wrapping the proximal segments of the newly regenerated primary dendrites, based on qualitative appearance (Fig. 4*G*, Extended Data Fig. 4-4*C*). Following pinch injury, we saw no obvious signs of direct injury to the glia (Fig. 4*G*, Extended Data Fig. 4-4*B*). On the spared side of the arbor, the glia appeared normal; on the pinched side of the arbor, the glia struggled to wrap the regenerated primary dendrites. The glia could, however, still wrap the axon and soma following pinch injury. Thus, glia appeared to not be directly damaged by either injury paradigm.

Lastly, we looked at the immune response following both injury paradigms. There are three types of immune cells (hemocytes) in *Drosophila*: plasmatocytes, crystal cells, and lamellocytes (Rizki, 1957; Rizki and Rizki, 1983, 1992; Tepass et al., 1994; Lebestky et al., 2000). Approximately 95% of all circulating hemocytes are plasmatocytes, and the remaining 5% are crystal cells. Lamellocytes are only stimulated by parasitism (Rizki and Rizki, 1992; Meister and Lagueux, 2003). While most hemocytes migrate through the hemolymph and have no specific localizations, in abdominal segments 1–7 (in which these injury experiments were performed), there are some groups of localized hemocytes in the epidermal-muscular pocket (Makhijani et al., 2011). Proper localization and survival of these resident hemocytes is supported by the da neurons (Makhijani et al., 2011).

These resident hemocytes include the dorsal vessel cluster (near the top of the ddaC class IV dendritic arbor), the dorsal stripe (near the soma), and the lateral patch (which extends from the bottom of the ddaC class IV dendritic arbor into the neighboring v'ada class IV arbor; Makhijani et al., 2011). These three patches of hemocytes were visible in uninjured hemisegments (yellow boxes in Extended Data Figs. 4-5*A*, 4-6*A*). Quantification of resident hemocytes near uninjured neurons indicated that there was no significant difference between the number of hemocytes on either side of the arbor or the total arbor at 24 or 72 h control (Extended Data Fig. 4-6*B*). Following 2p injury, there was no major accumulation of hemocytes near the regenerating arbor, and the three patches of hemocytes appeared normal (Fig. 4*H*, Extended Data Fig. 4-5*C*; yellow boxes in Extended Data Fig. 4-5*C* highlight the presence of normal resident hemocyte groups). Following pinch injury, however, there was major accumulation of hemocytes to the pinched hemisegment, on both spared and pinched sides of the ddaC class IV arbor (Fig. 4*H*; Extended Data Figs. 4-5*B*, 4-6*C*). There were no longer three discrete patches of hemocytes; instead, there was a large accumulation of hemocytes near the focal point of injury (Fig. 4*H*, Extended Data Fig. 4-5*B*). Quantification of the hemocyte influx into the pinched hemisegment showed that there was a significant increase in the number of hemocytes following pinch injury compared with uninjured neurons at both 24 and 72 h AI (Extended Data Fig. 4-6*E*), on both the pinched and spared half of the arbor or the total arbor area. When we looked at the count of hemocyte number over time, the number of hemocytes did not change from 24 to 72 h AI for the spared side of the pinched arbor or the total arbor (Extended Data Fig. 4-6*D*). There was a significant decrease in the number of hemocytes on the injured side of the pinched arbor from 24 to 72 h AI. The reduction of hemocytes over time on the injured side of a pinched arbor could indicate the start of a resolution of the immune response. Interestingly, resolution of an immune response following pinch injury has been reported to conclude by 24 h after wounding, which is faster than what we observed (Babcock et al., 2008; Burra et al., 2013). Overall, this influx of hemocytes to the injured hemisegment pointed toward a large immune reaction following the widespread tissue damage caused by pinch injury, representing another possible reason for poor dendrite regrowth into empty territory.

### Damage to the ECM and epidermis restrict dendrite regeneration

To demonstrate that the discrete regenerative responses observed following the half-pinch + half-2p assay (Fig. 3) were, in fact, due to injury-specific differences in the surrounding tissue environment, we repeated the half-pinch + half-2p assay with the ECM viking-GFP protein trap or the Armadillo-GFP epidermal marker (Fig. 5). Regenerative outcomes following half-pinch + half-2p injury with either extracellular marker mirrored the results seen in Figure 3, where there was substantially more dendrite regrowth into the empty territory on the side of the neuron where the 2p-injury had been performed (Fig. 5*A*,*C*). There was a significant increase in the area coverage of the 2p-injured half of the regenerating arbor at 72 h AI compared with the pinch-injured side of the arbor (Fig. 5*B*,*D*). Importantly, the extracellular damage—either to the ECM or epidermal cells—could be visualized and aligned with the pinch-injured side of the regenerating arbors. The ECM “scar” following pinch injury is highlighted with a white outline at 72 h AI and aligned perfectly with the congested dendrites attempting to regenerate into the damaged territory (Fig. 5*A*). There was a stark contrast between the damaged and misshapen epidermal cell morphology of the pinch-injured versus the intact, cobblestone morphology of the 2p-injured sides of the hemisegment (Fig. 5*C*). By combining the half-pinch + half-2p injuries with these extracellular markers, the discrete differences in regenerative capacity are directly linked to the damage sustained by the extracellular tissues due to pinch injury. These data reinforce our conclusion that damage to the extracellular environment must be considered to effectively evaluate neuronal regeneration in a real-world context.

## Discussion

Here, we have established a pinch injury method to injure dendrites that causes holistic tissue damage, effectively damaging the neurons and their dendrites, the ECM, and epidermis, while leaving the glia largely intact. This pinch injury method elicits a robust immune infiltration to the pinch-injured hemisegment. Importantly, the surrounding tissue damage restricts invasive dendrite regeneration into the empty territory, instead driving compensatory regeneration on the spared side of the arbor. This contrasts sharply with the invasive dendritic growth observed following 2p laser injury, where the epidermis and ECM remain undamaged. The discrete regenerative response to these two injury paradigms was further demonstrated in our half-pinch + half-2p injury paradigm: the pinched half of the class IV ddaC neuron exhibited reduced branch number, reduced branch length, and reduced area coverage compared with the 2p-injured half of the dendritic arbor. Performing the half-pinch + half-2p injury paradigm with the ECM and epidermal cell markers revealed that restricted dendrite regeneration spatially coincides with tissue damage, directly implicating the extracellular environment as a driver of poor regenerative outcomes. Given that the pinch injury damages not only neurons but also surrounding tissue, analogous to what occurs in traumatic brain injury (TBI), blunt force trauma, sports-related injuries, and blast injuries, this model provides a physiologically relevant platform to study neuronal regeneration (Hicks et al., 2010; Mustafa and Alshboul, 2013; George and Geller, 2018). In these real-world injuries, damage extends beyond neurons to include the ECM, supportive glial cells, and endothelial cells comprising the blood–brain barrier, making the pinch injury a more accurate model of acute neurotrauma than laser ablation alone (Gaudet et al., 2011; George and Geller, 2018; Yip et al., 2022).

The pinch injury method we describe here complements existing injury paradigms used to study neuronal regeneration. While laser ablation (either 2p or pulsed UV laser) has been instrumental in advancing our understanding of dendrite regeneration due to its precision and reproducibility (Duarte et al., 2024; Hertzler and Rolls, 2024; Prange et al., 2024; Hwu et al., 2025), it is fundamentally different from physiological injury in that surrounding tissue is left intact. Mechanical injury models such as axon crush injuries, commonly used to study peripheral nerve regeneration, cause widespread tissue damage similar to our pinch injury. Axon crush paradigms in *Drosophila*, vertebrate, and other invertebrate models have been widely used to study axon regeneration and have revealed that both the neuron and its local environment must be considered when evaluating regenerative capacity (von Bernhardi and Muller, 1995; Menorca et al., 2013; Rooney and Freeman, 2014; Akram et al., 2022; Bhattacharya, 2023; Pluta et al., 2025; Waller et al., 2025). Similarly, peripheral nerve injuries in mice, including sciatic nerve crush and pinch injuries, cause damage to neurons, Schwann cells, blood vessels, and ECM, triggering inflammatory responses that influence regenerative outcomes (Gaudet et al., 2011). Our dendrite pinch injury model parallels these axon injury paradigms but allows for spatial compartmentalization of damage to part of a single neuron. We liken this aspect of our pinch injury model to the novel twist injury, which uses a heat-pulled glass capillary tube to make a fine, hair-like structure which effectively severs one of the primary dendrites of PVD *C. elegans* neurons (Singh et al., 2026) or the removal of one first-order branch in a medicinal leech mechanosensory neuron, the dorsal pressure-sensitive neuron (*P_D_*; Wang and Macagno, 1998). This unique feature of our pinch injury model—injuring only half of the dendritic arbor—enables direct within-neuron comparisons of regeneration in damaged versus undamaged tissue environments, providing insights that are harder to glean from whole-nerve injury models.

The accumulation of the *Drosophila* homolog of collagen IV, viking (vkg), and damage to the ECM that we observed following pinch injury may represent a scar-like structure that impedes dendrite regeneration. This vkg/collagen IV accumulation and scar-like structure is similar to the collagenous scar that forms in the ECM following dorsal pinch injury in *Drosophila* larvae (Ramos-Lewis et al., 2018). Further, in mammalian spinal cord injury (SCI) and TBI, glial scars form at the lesion site, characterized by reactive astrocytes, the deposition of chondroitin sulfate proteoglycans (CSPGs), and ECM remodeling (Siddiqui et al., 2022; Tran et al., 2022). Similarly, fibrotic scars composed of collagen and fibronectin create physical and chemical barriers to axonal regeneration following nerve injury (Ayazi et al., 2022). The vkg/collagen IV accumulation we observe in the pinched tissue shares features with these injury-induced scars, raising the possibility that it serves as a barrier to dendrite regrowth. However, the mechanism by which this damaged ECM might restrict dendrite regeneration remains unclear. Injured dendrites might be actively repelled by molecular cues within the damaged tissue, analogous to how CSPGs inhibit axon growth in the glial scar through receptor-mediated signaling (Ohtake and Li, 2015). Alternatively, the damaged epidermis and disrupted ECM architecture may simply fail to provide the adhesive substrate or trophic support necessary for dendrite attachment and growth (Sharma et al., 2023). Distinguishing between active repulsion and failure of permissive attachment is critical for understanding whether dendrites avoid the injured territory or are unable to invade it. Our half-pinch + half-2p injury paradigm, which reveals preferential growth on the 2p-injured side, suggests that the lack of tissue damage may be sufficient to promote regeneration, favoring a model in which the damaged tissue fails to support dendrite growth rather than actively repelling it.

Irregularities in epidermal cell morphology following wounding in *Drosophila* larvae have been described before (Kwon et al., 2010). We speculate that the disfigured epidermal cells we observed were damaged by pinch injury and subsequently experienced epidermal cell fusion (White et al., 2026). In the larval epidermis, wounding can lead to epidermal cell fusion and the formation of multinucleate syncytial cells (Galko and Krasnow, 2004; Wang et al., 2015; White et al., 2026). Wounding the adult *Drosophila* epidermis also leads to syncytial formation along with polyploidization further from the wound's edge (Losick et al., 2013; Losick, 2016). This hypothesis of polyploidization is attractive given that polyploidization as a result of endoreplication is required for epidermal cells in *Drosophila* larvae, acting as nonprofessional phagocytes, to successfully clear sensory dendrite debris following injury (Han et al., 2014; Huang et al., 2025).

The tissue damage following pinch injury is not static but rather evolves over time, potentially creating an increasingly restrictive environment for dendrite regeneration. We observe that vkg/collagen IV accumulation in the damaged ECM increases progressively following injury. This temporal pattern suggests that the barriers to regeneration may intensify as time progresses, rather than resolving to create a more permissive substrate for growth. The dynamic remodeling of the ECM following injury may be mediated in part by matrix metalloproteinases (MMPs), which degrade ECM components and play complex, time-dependent roles in regeneration (Yong, 2005; Phillips et al., 2014). In mammalian CNS injury, MMPs are rapidly upregulated in the acute phase and contribute to both beneficial effects, such as clearing debris and allowing cell migration, and detrimental effects, including disruption of the blood–brain barrier and exacerbation of inflammation (Andries et al., 2017). As such, the accumulation of vkg/collagen IV that we observe may reflect an active remodeling and reorganization of the ECM by MMPs, which, in turn, could progressively limit the ability of the ECM to form a permissive substrate for dendrite growth (DeVault et al., 2018; Furusawa and Emoto, 2021).

The immune response following pinch injury is also a highly evolving process. Prior to injury, there are two subsets of hemocytes aside from the lymph gland. Sessile hemocytes are present in hematopoietic pockets between the larval epidermis and musculature (Márkus et al., 2009; Makhijani et al., 2011; 2017). The second set of hemocytes include those that circulate within the hemolymph (Babcock et al., 2008), and these two subsets of hemocyte populations can exchange themselves, transitioning between circulatory and sessile states (Honti et al., 2010; Makhijani et al., 2011). Those that circulate within the hemolymph are described to patrol the larval body and only “happen upon” a wound when they bump into it as they circulate. At that point, those hemocytes adhere and begin proliferating in response to the damage (Babcock et al., 2008). Once the damage and wound are resolved (for an epidermal pinch wound, this is ∼24 h after wounding), the hemocytes are released back into circulation by the healed epidermis (Babcock et al., 2008; Burra et al., 2013). Our hemocyte accumulation data indicates a more prolonged immune reaction, where there exists a robust presence of hemocytes up to at least 72 h after pinch injury. The hemocyte accumulation is beginning to be resolved by 72 h after injury on the pinched side but is still high and stable on the spared side of an injured hemisegment.

What might account for these observations about hemocyte retention? Long-lived chemoattractants might continuously recruit hemocytes, structural features might impede hemocyte migration, and/or injury-induced alterations in hemisegment size might complicate the data. We consider these possibilities in turn.

Various chemoattractants could be responsible for prolonged hemocyte recruitment. Upon initial epidermal wounding in *Drosophila* larvae, damage-associated molecular patterns (DAMPs) are produced, such as reactive oxygen species (ROS) like H_2_O_2_ (Razzell et al., 2013; Shaukat et al., 2015; Niethammer, 2016; Evans et al., 2022). Production of ROS following epidermal cell damage is required for hemocyte activation and proliferation (Moreira et al., 2010; Razzell et al., 2013; Evans et al., 2022). Although ROS are immediately produced upon wound formation and could initially stimulate hemocyte recruitment and proliferation, they are short-lived, so they are unlikely to account for long-term hemocyte retention (Niethammer et al., 2009). Long-lasting signals could include actinin-β and/or the Pvr/Pvf pathway. Actinin-β is expressed by the da neurons and maintains the hematopoietic clusters near da neurons through TGF-β signaling in the hemocytes (Makhijani et al., 2017). Pvr is a receptor tyrosine kinase in *Drosophila*, related to platelet-derived growth factor receptor and vascular endothelial growth factor receptor, which can bind to any of three ligands in *Drosophila*: *Pvf1*, *Pvf2*, and *Pvf3*. Pvr is expressed by epidermal cells, and proper epidermal wound healing and epidermal cell migration requires hemolymph-derived Pvf1 to bind to Pvr on the wounded epidermis (Wu et al., 2009). Pvr is also expressed by hemocytes, and Pvf1 binding to Pvr on hemocytes is required for hemocyte spreading at the wound and epidermal wound closure (Tsai et al., 2022). Pvr also plays a role in neuron morphology, as class IV ddaC neurons have reduced branch number and branch length in *Pvr*, *Pvf2*, or *Pvf3* mutants, and this signaling is important for class IV ddaC neuron function as larval nociceptors (Lopez-Bellido et al., 2019). Taking these data together, we can consider that pinch injury may induce Pvf ligand expression or release, activating Pvr on epidermal cells, hemocytes, and/or neurons. This could in turn promote prolonged hemocyte spreading at the epidermal wound, explaining our persistent hemocyte presence (Extended Data Fig. 4-6). It could also promote epidermal wound closure and healing following pinch injury and additionally be important for maintenance of the function of the remaining arbor of class IV ddaC neurons following pinch injury. The decrease in hemocyte number on the pinched half of the arbor could alternatively be a reflection of the respective sizes of each side of a pinched arbor. Due to the damage by pinch injury, the width of the injured side of a pinched arbor is smaller than that of the spared side. A decrease in physical space over time, as a result of repairing surrounding tissue following pinch injury, could reduce the number of hemocytes that could “fit” into the damaged territory. Finally, the damage to the ECM and the formation of a “scar” could impede hemocyte migration into the damaged half of the hemisegment, as *lamininB* mutants experience decreased hemocyte motility (Korabel et al., 2022).

While we have identified damage to the epidermis and ECM as key features of the pinch injury, the full extent of tissue damage and the cellular sources of ECM remodeling remain to be fully characterized. We used the Gal4^A58^ line to visualize vkg/collagen IV accumulation following injury, and while Gal4^A58^ is expressed in epidermal cells, it is also expressed in the fat body (Ramos-Lewis et al., 2018). Therefore, the vkg/collagen IV accumulation we observe may derive not only from epidermal cells but also from the fat body, which serves as a major source of ECM proteins and circulating factors in *Drosophila* (Rodriguez et al., 1996; Isabella and Horne-Badovinac, 2015; Ramos-Lewis et al., 2018). Distinguishing between these sources will be important for understanding the origin and regulation of the ECM response to injury. Moreover, vkg/collagen IV is just one of many ECM components, which we selected due to its high abundance and structural importance (Rozario and DeSimone, 2010). Other ECM components, including laminins, proteoglycans, and other collagens, are likely also disrupted or dysregulated following pinch injury. The complete ECM composition in the injured territory, and how it differs from uninjured tissue, may provide insight into what makes the environment restrictive for dendrite growth. The pinch injury damages all cells within the compressed tissue, not only the sensory neuron and individual other cell types we imaged here. It is possible we are damaging or killing other da neurons and cell types that normally provide trophic support, guidance cues, or other proregenerative signals that facilitate dendritic outgrowth. Uncharacterized aspects of the injury underscore the complexity of the tissue damage and highlight the need for comprehensive characterization.

Lastly, an important consideration is how the combined damage to neurons and surrounding tissue would affect sensory function. Both class IV da neurons and epidermal cells are mechanosensitive and work cooperatively to detect mechanical stimuli in *Drosophila* larvae (Luedke et al., 2024; Yoshino et al., 2025). Damage as we observe following pinch injury could compromise mechanosensation through multiple mechanisms, including direct loss of epidermal mechanosensory function and disruption of neuron-epidermis communication (Yang and Chien, 2019; Luedke et al., 2024; Yoshino et al., 2025). This has important implications for understanding recovery from injury in clinical cases, where damage to neurons, glia, and other supporting cell types occurs simultaneously. Even if dendrites successfully grow new branches after pinch injury, as we observed through compensatory growth on the uninjured side of the arbor, full restoration of sensory function might require neuronal regrowth as well as repair of epidermal cells and the re-establishment of neuron–epidermis interactions.

## Synthesis

Reviewing Editor: Fabienne Poulain, University of South Carolina Decisions are customarily a result of the Reviewing Editor and the peer reviewers coming together and discussing their recommendations until a consensus is reached. When revisions are invited, a fact-based synthesis statement explaining their decision and outlining what is needed to prepare a revision will be listed below. The following reviewer(s) agreed to reveal their identity: NONE.

In this manuscript, the authors provide a new experimental approach to study dendrite regrowth after injury in the Drosophila larval somatosensory system. Prior studies used a two-photon approach to sever dendritic arbors and assess dendrite regrowth. However, this approach does not affect the local environment of the neuron and as such, does not model realistic injuries that affect neighboring tissues. Here, using a novel pinch injury method, the authors damage the neuron asymmetrically, severing the dendrites and surrounding tissue (including epidermal cells, glial cells and extracellular matrix) on one side of an arbor and leaving the other unscathed. They found that the pinch injury alters substrate epidermal cells and immune cell distribution, leads to the accumulation of the ECM component collagen IV, and prevents dendritic regrowth, with dendrites preferentially regenerating into areas of intact tissues. Altogether, this is a well-presented description of a mechanical neuronal injury paradigm that is likely to be of broad interest and sets the stage for future studies on the contributions of environmental damages to neuronal regeneration. It is appropriate for publication in eNeuro, provided the following points are addressed.

Major points:

1. The authors state that the pinch induced damage is reproducible, but in contrast to the 2P approach, the damage induced by pinch could be very user dependent, even though the video recording of the pinch assay may help somewhat with this. The authors describe "minimal pressure" as sufficient to cause damage. To enhance reproducibility, the authors could perhaps report the range of effects seen with "maximal" and "medium" pressure, so that others could calibrate what minimal pressure is based on the effects they observe.

2. The authors perform CaMPARI imaging to show that pinching induces Ca influx into damaged class IV neurons, which is proposed to be an initial step in injury detection. However, since these are nociceptors, the Ca response could be a part of the normal sensory responses of these cells rather than related to injury. Does Ca accumulate as well in non-nociceptive neurons that are damaged by the pinch?

3. The authors claim that branches are added to the uninjured half of the arbor. However, the quantification appears to represent the total number of branches (and dendrite length, etc.) of the whole arbor (e.g. see Figure 2). Either the authors should clarify what is being quantified in the graphs, or they should break up their quantification to separate out the two halves of the arbor (injured and uninjured).

4. The epidermal cell data was very difficult to interpret. In the images in Figure 4, it was difficult to discern the individual epidermal cells and whether and how they were affected. It would be helpful to show pre- and post-injury images for an individual animal to illustrate the effect of the injury on epidermal cells. In their current images, the authors do not quantify epidermal cell morphology or highlight example(s) of disrupted epidermal cells to reflect their claims in the text (lines 489-492).

5. The authors claim that the regenerating dendrites grow into territory surrounded by intact tissue. This claim, however, is not quantitatively supported (and is also difficult to qualitatively discern from the images). In other experiments, the authors use fluorescent markers of the ECM and epithelial cells; it would be helpful to use these markers to support the claim that dendrites preferentially extend into intact epithelial territories.

6. The authors claim that there is an influx of hemocytes into the territory damaged by the pinch. However, the hemocyte data is not quantified. The authors need to quantify the hemocytes in control and injured animals, and, ideally, determine the correlation between the hemocyte distribution and the pinch-induced damage.

Minor points:

1. Some of the images and anatomy may be unfamiliar to a broader audience. The authors are encouraged to add arrows and arrowheads to draw the attention of readers to areas of interest in the figure panels. One example of this is 4-3C, where readers must sort out the smaller epidermal cells and longer ones without any guidance.

2. In figure 4-2D, it is not clear why the authors change to dark and light green when they are quantifying the magenta channel in the neighboring image.

3. In several figures the authors label the uninjured controls as "X hrs AI", which is confusing since there is no injury in these images and plots. For example in Figure 2, both images and plots follow this convention. Could the authors use "x hrs control" instead?

4. The authors should be careful not to make claims about data that is not quantified. For example, the authors state (lines 506-507) that "the glia have trouble wrapping the proximal segments of the newly regenerated primary dendrites (Figure 4G and Figure 4-4C)," but these data are not quantified. The authors should make it clear that this is speculation based on a qualitative evaluation.

5. It could be helpful for the authors to highlight the pinched side in their figures with a bracket or bar. In later figures, which include different channels, it would help the reader to identify where the pinch occurred.

6. Based on the methods, it seems that two neurons are likely affected in each pinch since the pinch occurs over two hemi-segments. The authors should specific how many neurons are affected during each pinch.

7. The authors should specify how they quantified the CaMPARI signal, especially as they are using GFP to mark the neuronal morphology.

8. Line 478: "coracle" likely refers to a protein, in which case the first letter should be capitalized.

## References

[B1] Akram R, Anwar H, Javed MS, Rasul A, Imran A, Malik SA, Raza C, Khan IU, Sajid F, Iman T (2022) Axonal regeneration: underlying molecular mechanisms and potential therapeutic targets. Biomedicines 10:3186. 10.3390/biomedicines10123186 PMC9775075 36551942

[B2] Andries L, Van Hove I, Moons L, De Groef L (2017) Matrix metalloproteinases during axonal regeneration, a multifactorial role from start to finish. Mol Neurobiol 54:2114–2125. 10.1007/s12035-016-9801-x 26924318

[B3] Ayazi M, Zivkovic S, Hammel G, Stefanovic B, Ren Y (2022) Fibrotic scar in CNS injuries: from the cellular origins of fibroblasts to the molecular processes of fibrotic scar formation. Cells 11:2371. 10.3390/cells11152371 PMC9367779 35954214

[B4] Babcock DT, Brock AR, Fish GS, Wang Y, Perrin L, Krasnow MA, Galko MJ (2008) Circulating blood cells function as a surveillance system for damaged tissue in Drosophila larvae. Proc Natl Acad Sci U S A 105:10017–10022. 10.1073/pnas.0709951105 PMC2474562 18632567

[B5] Barolo S, Castro B, Posakony JW (2004) New Drosophila transgenic reporters: insulated P-element vectors expressing fast-maturing RFP. BioTechniques 36:436–442. 10.2144/04363ST03 15038159

[B6] Bhattacharya MR (2023) A nerve-wracking buzz: lessons from Drosophila models of peripheral neuropathy and axon degeneration. Front Aging Neurosci 15:1166146. 10.3389/fnagi.2023.1166146 PMC10442544 37614471

[B7] Brar HK, Dey S, Bhardwaj S, Pande D, Singh P, Dey S, Ghosh-Roy A (2022) Dendrite regeneration in C. elegans is controlled by the RAC GTPase CED-10 and the RhoGEF TIAM-1. PLoS Genet 18:e1010127. 10.1371/journal.pgen.1010127 PMC8989329 35344539

[B8] Burra S, Wang Y, Brock AR, Galko MJ (2013) Using *Drosophila* larvae to study epidermal wound closure and inflammation. In: Wound regeneration and repair (Gourdie R, Myers T, eds), Vol. 1037, pp 449–461. Totowa, NJ: Humana Press. 10.1007/978-1-62703-505-7_26 PMC3985134 24029952

[B9] Cheng LE, Song W, Looger LL, Jan LY, Jan YN (2010) The role of the TRP channel NompC in Drosophila larval and adult locomotion. Neuron 67:373–380. 10.1016/j.neuron.2010.07.004 PMC2933178 20696376

[B10] Cox RT, Kirkpatrick C, Peifer M (1996) Armadillo is required for adherens junction assembly, cell polarity, and morphogenesis during Drosophila embryogenesis. J Cell Biol 134:133–148. 10.1083/jcb.134.1.133 PMC2120919 8698810

[B11] Dansie LE, Ethell IM (2011) Casting a net on dendritic spines: the extracellular matrix and its receptors. Dev Neurobiol 71:956–981. 10.1002/dneu.20963 PMC3192312 21834084

[B12] DeVault L, Li T, Izabel S, Thompson-Peer KL, Jan LY, Jan YN (2018) Dendrite regeneration of adult Drosophila sensory neurons diminishes with aging and is inhibited by epidermal-derived matrix metalloproteinase 2. Genes Dev 32:402–414. 10.1101/gad.308270.117 PMC5900713 29563183

[B13] Duarte VN, Lam VT, Rimicci DS, Thompson-Peer KL (2024) Calcium plays an essential role in early-stage dendrite injury detection. Front Neurobiol 239:102635. 10.1016/j.pneurobio.2024.102635 PMC11305834 38825174

[B14] Evans CJ, Liu T, Girard JR, Banerjee U (2022) Injury-induced inflammatory signaling and hematopoiesis in Drosophila. Proc Natl Acad Sci U S A 119:e2119109119. 10.1073/pnas.2119109119 PMC8944666 35286208

[B15] Fosque BF, Sun Y, Dana H, Yang C-T, Ohyama T, Tadross MR, Patel R, Zlatic M, Kim DS, Ahrens MB (2015) Labeling of active neural circuits in vivo with designed calcium integrators. Science 347:755–760. 10.1126/science.1260922 25678659

[B16] Furusawa K, Emoto K (2021) Scrap and build for functional neural circuits: spatiotemporal regulation of dendrite degeneration and regeneration in neural development and disease. Front Cell Neurosci 14:613320. 10.3389/fncel.2020.613320 PMC7829185 33505249

[B17] Galko MJ, Krasnow MA (2004) Cellular and genetic analysis of wound healing in Drosophila larvae. PLoS Biol 2:e239. 10.1371/journal.pbio.0020239 PMC479041 15269788

[B18] Gaudet AD, Popovich PG, Ramer MS (2011) Wallerian degeneration: gaining perspective on inflammatory events after peripheral nerve injury. J Neuroinflammation 8:110. 10.1186/1742-2094-8-110 PMC3180276 21878126

[B19] George N, Geller HM (2018) Extracellular matrix and traumatic brain injury. J Neurosci Res 96:573–588. 10.1002/jnr.24151 PMC5803383 29344975

[B20] Grueber WB, Jan LY, Jan YN (2002) Tiling of the Drosophila epidermis by multidendritic sensory neurons. Development 129:2867–2878. 10.1242/dev.129.12.2867 12050135

[B21] Grueber WB, Jan LY, Jan YN (2003) Different levels of the homeodomain protein cut regulate distinct dendrite branching patterns of Drosophila multidendritic neurons. Cell 112:805–818. 10.1016/S0092-8674(03)00160-0 12654247

[B22] Han C, Jan LY, Jan Y-N (2011) Enhancer-driven membrane markers for analysis of nonautonomous mechanisms reveal neuron–glia interactions in Drosophila. Proc Natl Acad Sci U S A 108:9673–9678. 10.1073/pnas.1106386108 PMC3111288 21606367

[B23] Han C, Wang D, Soba P, Zhu S, Lin X, Jan LY, Jan Y-N (2012) Integrins regulate repulsion-mediated dendritic patterning of Drosophila sensory neurons by restricting dendrites in a 2D space. Neuron 73:64–78. 10.1016/j.neuron.2011.10.036 PMC3269260 22243747

[B24] Han C, Song Y, Xiao H, Wang D, Franc NC, Jan LY, Jan Y-N (2014) Epidermal cells are the primary phagocytes in the fragmentation and clearance of degenerating dendrites in Drosophila. Neuron 81:544–560. 10.1016/j.neuron.2013.11.021 PMC3995171 24412417

[B25] He L, Gulyanon S, Skanata MM, Karagyozov D, Heckscher ES, Krieg M, Tsechpenakis G, Gershow M, Tracey WD (2019) Direction selectivity in Drosophila proprioceptors requires the mechanosensory channel Tmc. Curr Biol 29:945–956.e943. 10.1016/j.cub.2019.02.025 PMC6884367 30853433

[B26] Heiman MG, Bülow HE (2024) Dendrite morphogenesis in Caenorhabditis elegans. Genetics 227:iyae056. 10.1093/genetics/iyae056 PMC11151937 38785371

[B27] Hertzler JI, Bernard AR, Rolls MM (2023) Dendrite regeneration mediates functional recovery after complete dendrite removal. Dev Biol 497:18–25. 10.1016/j.ydbio.2023.03.001 PMC10073339 36870669

[B28] Hertzler JI, Teng J, Bernard AR, Stone MC, Kline HL, Mahata G, Kumar N, Rolls MM (2024) Voltage-gated calcium channels act upstream of adenylyl cyclase Ac78C to promote timely initiation of dendrite regeneration. PLoS Genet 20:e1011388. 10.1371/journal.pgen.1011388 PMC11379402 39186815

[B29] Hertzler JI, Rolls MM (2024) Out with the old, in with the new: dendrite degeneration and regeneration. In: *Wiring the nervous system: mechanisms of axonal and dendritic remodelling in health and disease* (Tran TS, Yaron A, eds), Chapter 3, NY: River Publishers. 38536946

[B30] Hicks RR, Fertig SJ, Desrocher RE, Koroshetz WJ, Pancrazio JJ (2010) Neurological effects of blast injury. J Trauma Acute Care Surg 68:1257–1263. 10.1097/TA.0b013e3181d8956d PMC2958428 20453776

[B31] Honti V, Csordás G, Márkus R, Kurucz É, Jankovics F, Andó I (2010) Cell lineage tracing reveals the plasticity of the hemocyte lineages and of the hematopoietic compartments in Drosophila melanogaster. Mol Immunol 47:1997–2004. 10.1016/j.molimm.2010.04.017 20483458

[B32] Huang Y-C, Almeida Machado Costa C, Vergara Ruiz N, Wang X, Jevitt A, Breneman CM, Han C, Deng W-M (2025) Polyploidy promotes transformation of epithelial cells into nonprofessional phagocytes. Proc Natl Acad Sci U S A 122:e2427293122. 10.1073/pnas.2427293122 PMC12318187 40694325

[B33] Hughes CL, Thomas JB (2007) A sensory feedback circuit coordinates muscle activity in Drosophila. Mol Cell Neurosci 35:383–396. 10.1016/j.mcn.2007.04.001 PMC1978164 17498969

[B34] Hwang RY, Zhong L, Xu Y, Johnson T, Zhang F, Deisseroth K, Tracey WD (2007) Nociceptive neurons protect Drosophila larvae from parasitoid wasps. Curr Biol 17:2105–2116. 10.1016/j.cub.2007.11.029 PMC2225350 18060782

[B35] Hwu PT, Kim LA, Wood MA, Thompson-Peer KL (2025) In-vivo dendrite injury drives local mitochondrial contraction and dendrite branching. bioRxiv. 10.1101/2025.06.05.658131

[B36] Isabella AJ, Horne-Badovinac S (2015) Building from the ground up: basement membranes in Drosophila development. Curr Top Membr 76:305–336. 10.1016/bs.ctm.2015.07.001 PMC4772872 26610918

[B37] Jenett A, Rubin GM, Ngo T-T, Shepherd D, Murphy C, Dionne H, Pfeiffer BD, Cavallaro A, Hall D, Jeter J (2012) A GAL4-driver line resource for Drosophila neurobiology. Cell Rep 2:991–1001. 10.1016/j.celrep.2012.09.011 PMC3515021 23063364

[B38] Jiang N, Soba P, Parker E, Kim CC, Parrish JZ (2014) The microRNA bantam regulates a developmental transition in epithelial cells that restricts sensory dendrite growth. Development 141:2657–2668. 10.1242/dev.107573 PMC4067962 24924190

[B39] Kim ME, Shrestha BR, Blazeski R, Mason CA, Grueber WB (2012) Integrins establish dendrite-substrate relationships that promote dendritic self-avoidance and patterning in Drosophila sensory neurons. Neuron 73:79–91. 10.1016/j.neuron.2011.10.033 PMC3470655 22243748

[B40] Klapka N, Müller HW (2006) Collagen matrix in spinal cord injury. J Neurotrauma 23:422–436. 10.1089/neu.2006.23.422 16629627

[B41] Korabel N, Clemente GD, Han D, Feldman F, Millard TH, Waigh TA (2022) Hemocytes in Drosophila melanogaster embryos move via heterogeneous anomalous diffusion. Commun Phys 5:269. 10.1038/s42005-022-01051-6

[B42] Kwon Y-C, Baek SH, Lee H, Choe K-M (2010) Nonmuscle myosin II localization is regulated by JNK during Drosophila larval wound healing. Biochem Biophys Res Commun 393:656–661. 10.1016/j.bbrc.2010.02.047 20153725

[B43] Lebestky T, Chang T, Hartenstein V, Banerjee U (2000) Specification of Drosophila hematopoietic lineage by conserved transcription factors. Science 288:146–149. 10.1126/science.288.5463.146 10753120

[B44] Lesch C, Jo J, Wu Y, Fish GS, Galko MJ (2010) A targeted UAS-RNAi screen in Drosophila larvae identifies wound closure genes regulating distinct cellular processes. Genetics 186:943–957. 10.1534/genetics.110.121822 PMC2975283 20813879

[B45] Levy AD, Omar MH, Koleske AJ (2014) Extracellular matrix control of dendritic spine and synapse structure and plasticity in adulthood. Front Neuroanat 8:116. 10.3389/fnana.2014.00116 PMC4202714 25368556

[B46] Liesi P, Kauppila T (2002) Induction of type IV collagen and other basement-membrane-associated proteins after spinal cord injury of the adult rat may participate in formation of the glial scar. Exp Neurol 173:31–45. 10.1006/exnr.2001.7800 11771937

[B47] Long KR, Huttner WB (2019) How the extracellular matrix shapes neural development. R Soc Open Biol 9:180216. 10.1098/rsob.180216 PMC6367132 30958121

[B48] Lopez-Bellido R, Puig S, Huang PJ, Tsai C-R, Turner HN, Galko MJ, Gutstein HB (2019) Growth factor signaling regulates mechanical nociception in flies and vertebrates. J Neurosci 39:6012–6030. 10.1523/JNEUROSCI.2950-18.2019 PMC6650988 31138657

[B49] Losick VP, Fox DT, Spradling AC (2013) Polyploidization and cell fusion contribute to wound healing in the adult Drosophila epithelium. Curr Biol 23:2224–2232. 10.1016/j.cub.2013.09.029 PMC3898104 24184101

[B50] Losick VP (2016) Wound-induced polyploidy is required for tissue repair. Adv Wound Care 5:271–278. 10.1089/wound.2014.0545 PMC4876532 27274437

[B51] Luedke KP, Yoshino J, Yin C, Jiang N, Huang JM, Huynh K, Parrish JZ (2024) Dendrite intercalation between epidermal cells tunes nociceptor sensitivity to mechanical stimuli in Drosophila larvae. PLoS Genet 20:e1011237. 10.1371/journal.pgen.1011237 PMC11075839 38662763

[B52] Makhijani K, Alexander B, Tanaka T, Rulifson E, Brückner K (2011) The peripheral nervous system supports blood cell homing and survival in the Drosophila larva. Development 138:5379–5391. 10.1242/dev.067322 PMC3222213 22071105

[B53] Makhijani K, et al. (2017) Regulation of Drosophila hematopoietic sites by activin-β from active sensory neurons. Nat Commun 8:15990. 10.1038/ncomms15990 PMC5537569 28748922

[B54] Márkus R, Laurinyecz B, Kurucz É, Honti V, Bajusz I, Sipos B, Somogyi K, Kronhamn J, Hultmark D, Andó I (2009) Sessile hemocytes as a hematopoietic compartment in Drosophila melanogaster. Proc Natl Acad Sci U S A 106:4805–4809. 10.1073/pnas.0801766106 PMC2660760 19261847

[B55] Meister M, Lagueux M (2003) Drosophila blood cells. Cell Microbiol 5:573–580. 10.1046/j.1462-5822.2003.00302.x 12925127

[B56] Menorca RM, Fussell TS, Elfar JC (2013) Peripheral nerve trauma: mechanisms of injury and recovery. Hand Clin 29:317. 10.1016/j.hcl.2013.04.002 PMC4408553 23895713

[B57] Moeyaert B, Holt G, Madangopal R, Perez-Alvarez A, Fearey BC, Trojanowski NF, Ledderose J, Zolnik TA, Das A, Patel D (2018) Improved methods for marking active neuron populations. Nat Commun 9:4440. 10.1038/s41467-018-06935-2 PMC6202339 30361563

[B58] Moreira S, Stramer B, Evans I, Wood W, Martin P (2010) Prioritization of competing damage and developmental signals by migrating macrophages in the Drosophila embryo. Curr Biol 20:464–470. 10.1016/j.cub.2010.01.047 20188558

[B59] Morin X, Daneman R, Zavortink M, Chia W (2001) A protein trap strategy to detect GFP-tagged proteins expressed from their endogenous loci in Drosophila. Proc Natl Acad Sci U S A 98:15050–15055. 10.1073/pnas.261408198 PMC64981 11742088

[B60] Mustafa AG, Alshboul OA (2013) Pathophysiology of traumatic brain injury. Neurosci J 18:222–235. 10.17712/1658-3183.2021 23887212

[B62] Niethammer P (2016) The early wound signals. Curr Opin Genet Dev 40:17–22. 10.1016/j.gde.2016.05.001 PMC5278878 27266971

[B61] Niethammer P, Grabher C, Look AT, Mitchison TJ (2009) A tissue-scale gradient of hydrogen peroxide mediates rapid wound detection in zebrafish. Nature 459:996–999. 10.1038/nature08119 PMC2803098 19494811

[B63] Nye DM, Albertson RM, Weiner AT, Hertzler JI, Shorey M, Goberdhan DC, Wilson C, Janes KA, Rolls MM (2020) The receptor tyrosine kinase Ror is required for dendrite regeneration in Drosophila neurons. PLoS Biol 18:e3000657. 10.1371/journal.pbio.3000657 PMC7067388 32163406

[B64] Ohtake Y, Li S (2015) Molecular mechanisms of scar-sourced axon growth inhibitors. Brain Res 1619:22–35. 10.1016/j.brainres.2014.08.064 PMC4345149 25192646

[B65] Oren-Suissa M, Gattegno T, Kravtsov V, Podbilewicz B (2017) Extrinsic repair of injured dendrites as a paradigm for regeneration by fusion in Caenorhabditis elegans. Genetics 206:215–230. 10.1534/genetics.116.196386 PMC5419471 28283540

[B66] Orsulic S, Peifer M (1996) An in vivo structure-function study of armadillo, the beta-catenin homologue, reveals both separate and overlapping regions of the protein required for cell adhesion and for wingless signaling. J Cell Biol 134:1283–1300. 10.1083/jcb.134.5.1283 PMC2120977 8794868

[B67] Parrish JZ, Xu P, Kim CC, Jan LY, Jan YN (2009) The microRNA bantam functions in epithelial cells to regulate scaling growth of dendrite arbors in Drosophila sensory neurons. Neuron 63:788–802. 10.1016/j.neuron.2009.08.006 PMC2772869 19778508

[B68] Paveliev M, Fenrich KK, Kislin M, Kuja-Panula J, Kulesskiy E, Varjosalo M, Kajander T, Mugantseva E, Ahonen-Bishopp A, Khiroug L (2016) HB-GAM (pleiotrophin) reverses inhibition of neural regeneration by the CNS extracellular matrix. Sci Rep 6:33916. 10.1038/srep33916 PMC5037378 27671118

[B69] Peterson SL, Benowitz LI (2018) Mammalian dendritic regrowth: a new perspective on neural repair. Brain 141:1891–1894. 10.1093/brain/awy165 PMC6658712 30053179

[B70] Phillips LL, Chan JL, Doperalski AE, Reeves TM (2014) Time dependent integration of matrix metalloproteinases and their targeted substrates directs axonal sprouting and synaptogenesis following central nervous system injury. Neural Regen Res 9:362–376. 10.4103/1673-5374.128237 PMC4146196 25206824

[B71] Pluta NA, Gaviria M, Sabbag CM, Hill S (2025) Advancements in peripheral nerve injury research using lab animals. Anatomia 4:8. 10.3390/anatomia4020008

[B72] Poe AR, Tang L, Wang B, Li Y, Sapar ML, Han C (2017) Dendritic space-filling requires a neuronal type-specific extracellular permissive signal in Drosophila. Proc Natl Acad Sci U S A 114:E8062–E8071. 10.1073/pnas.1707467114 PMC5617288 28874572

[B73] Prange SE, Bhakta IN, Sysoeva D, Jean GE, Madisetti A, Le HH, Duong LU, Hwu PT, Melton JG, Thompson-Peer KL (2024) Dendrite injury triggers neuroprotection in Drosophila models of neurodegenerative disease. Sci Rep 14:24766. 10.1038/s41598-024-74670-4 PMC11494097 39433621

[B74] Ramos-Lewis W, LaFever KS, Page-McCaw A (2018) A scar-like lesion is apparent in basement membrane after wound repair in vivo. Matrix Biol 74:101–120. 10.1016/j.matbio.2018.07.004 PMC6250587 29981372

[B75] Razzell W, Evans IR, Martin P, Wood W (2013) Calcium flashes orchestrate the wound inflammatory response through DUOX activation and hydrogen peroxide release. Curr Biol 23:424–429. 10.1016/j.cub.2013.01.058 PMC3629559 23394834

[B76] Rizki TM (1957) Alterations in the haemocyte population of Drosophila melanogaster. J Morphol 100:437–458. 10.1002/jmor.1051000303

[B77] Rizki TM, Rizki RM (1983) Blood cell surface changes in Drosophila mutants with melanotic tumors. Science 220:73–75. 10.1126/science.6402819 6402819

[B78] Rizki TM, Rizki RM (1992) Lamellocyte differentiation in Drosophila larvae parasitized by Leptopilina. Dev Comp Immunol 16:103–110. 10.1016/0145-305X(92)90011-Z 1499832

[B79] Rodriguez A, Zhou Z, Tang ML, Meller S, Chen J, Bellen H, Kimbrell DA (1996) Identification of immune system and response genes, and novel mutations causing melanotic tumor formation in Drosophila melanogaster. Genetics 143:929–940. 10.1093/genetics/143.2.929 PMC1207349 8725239

[B80] Rooney TM, Freeman MR (2014) Drosophila models of neuronal injury. ILAR J 54:291–295. 10.1093/ilar/ilt057 PMC3962260 24615442

[B81] Rozario T, DeSimone DW (2010) The extracellular matrix in development and morphogenesis: a dynamic view. Dev Biol 341:126–140. 10.1016/j.ydbio.2009.10.026 PMC2854274 19854168

[B82] Schindelin J, Arganda-Carreras I, Frise E, Kaynig V, Longair M, Pietzsch T, Preibisch S, Rueden C, Saalfeld S, Schmid B (2012) Fiji: an open-source platform for biological-image analysis. Nat Methods 9:676–682. 10.1038/nmeth.2019 PMC3855844 22743772

[B83] Sepp KJ, Schulte J, Auld VJ (2001) Peripheral glia direct axon guidance across the CNS/PNS transition zone. Dev Biol 238:47–63. 10.1006/dbio.2001.0411 11783993

[B84] Sharma A, Hill KE, Schwarzbauer JE (2023) Extracellular matrix composition affects outgrowth of dendrites and dendritic spines on cortical neurons. Front Cell Neurosci 17:1177663. 10.3389/fncel.2023.1177663 PMC10300442 37388410

[B85] Shaukat Z, Liu D, Gregory S (2015) Sterile inflammation in Drosophila. Mediators Inflamm 2015:369286. 10.1155/2015/369286 PMC4408615 25948885

[B86] Siddiqui N, Oshima K, Hippensteel JA (2022) Proteoglycans and glycosaminoglycans in central nervous system injury. Am J Physiol Cell Physiol 323:C46–C55. 10.1152/ajpcell.00053.2022 PMC9273265 35613357

[B87] Singh P, Vasudevan M, Dey S, Selvarasu K, Balakrishnan S, Bassi D, Ghosh-Roy A (2026) The conserved fibroblast growth factor receptor–based signaling is required for dendrite regeneration. Proc Natl Acad Sci U S A 123:e2506886123. 10.1073/pnas.2506886123 PMC13016080 41849383

[B88] Song W, Onishi M, Jan LY, Jan YN (2007) Peripheral multidendritic sensory neurons are necessary for rhythmic locomotion behavior in Drosophila larvae. Proc Natl Acad Sci U S A 104:5199–5204. 10.1073/pnas.0700895104 PMC1820883 17360325

[B89] Song Y, Ori-McKenney KM, Zheng Y, Han C, Jan LY, Jan YN (2012) Regeneration of Drosophila sensory neuron axons and dendrites is regulated by the Akt pathway involving Pten and microRNA bantam. Genes Dev 26:1612–1625. 10.1101/gad.193243.112 PMC3404388 22759636

[B90] Stevens LJ, Page-McCaw A (2012) A secreted MMP is required for reepithelialization during wound healing. Mol Biol Cell 23:1068–1079. 10.1091/mbc.e11-09-0745 PMC3302734 22262460

[B91] Stone MC, Roegiers F, Rolls MM (2008) Microtubules have opposite orientation in axons and dendrites of Drosophila neurons. Mol Biol Cell 19:4122–4129. 10.1091/mbc.e07-10-1079 PMC2555934 18667536

[B92] Stone MC, Albertson RM, Chen L, Rolls MM (2014) Dendrite injury triggers DLK-independent regeneration. Cell Rep 6:247–253. 10.1016/j.celrep.2013.12.022 PMC3954604 24412365

[B93] Stone MC, Seebold DY, Shorey M, Kothe GO, Rolls MM (2022) Dendrite regeneration in the vertebrate spinal cord. Dev Biol 488:114–119. 10.1016/j.ydbio.2022.05.014 PMC10046145 35644253

[B94] Stramer B, Wood W, Galko MJ, Redd MJ, Jacinto A, Parkhurst SM, Martin P (2005) Live imaging of wound inflammation in Drosophila embryos reveals key roles for small GTPases during in vivo cell migration. J Cell Biol 168:567–573. 10.1083/jcb.200405120 PMC2171743 15699212

[B95] Sugimura K, Yamamoto M, Niwa R, Satoh D, Goto S, Taniguchi M, Hayashi S, Uemura T (2003) Distinct developmental modes and lesion-induced reactions of dendrites of two classes of Drosophila sensory neurons. J Neurosci 23:3752–3760. 10.1523/JNEUROSCI.23-09-03752.2003 PMC6742166 12736346

[B96] Tenenbaum CM, Misra M, Alizzi RA, Gavis ER (2017) Enclosure of dendrites by epidermal cells restricts branching and permits coordinated development of spatially overlapping sensory neurons. Cell Rep 20:3043–3056. 10.1016/j.celrep.2017.09.001 PMC5662031 28954223

[B97] Tepass U, Fessler LI, Aziz A, Hartenstein V (1994) Embryonic origin of hemocytes and their relationship to cell death in Drosophila. Development 120:1829–1837. 10.1242/dev.120.7.1829 7924990

[B98] Terada S-I, Matsubara D, Onodera K, Matsuzaki M, Uemura T, Usui T (2016) Neuronal processing of noxious thermal stimuli mediated by dendritic Ca2+ influx in Drosophila somatosensory neurons. eLife 5:e12959. 10.7554/eLife.12959 PMC4786431 26880554

[B99] Thompson-Peer KL, DeVault L, Li T, Jan LY, Jan YN (2016) In vivo dendrite regeneration after injury is different from dendrite development. Genes Dev 30:1776–1789. 10.1101/gad.282848.116 PMC5002981 27542831

[B100] Tran AP, Warren PM, Silver J (2022) New insights into glial scar formation after spinal cord injury. Cell Tissue Res 387:319–336. 10.1007/s00441-021-03477-w PMC8975767 34076775

[B101] Tsai C-R, Anderson AE, Burra S, Jo J, Galko MJ (2017) Yorkie regulates epidermal wound healing in Drosophila larvae independently of cell proliferation and apoptosis. Dev Biol 427:61–71. 10.1016/j.ydbio.2017.05.006 PMC5713895 28514643

[B102] Tsai C-R, Wang Y, Jacobson A, Sankoorikkal N, Chirinos JD, Burra S, Makthal N, Kumaraswami M, Galko MJ (2022) Pvr and distinct downstream signaling factors are required for hemocyte spreading and epidermal wound closure at Drosophila larval wound sites. G3 12:jkab388. 10.1093/g3journal/jkab388 PMC8728012 34751396

[B103] Van De Bor V, Zimniak G, Papone L, Cerezo D, Malbouyres M, Juan T, Ruggiero F, Noselli S (2015) Companion blood cells control ovarian stem cell niche microenvironment and homeostasis. Cell Rep 13:546–560. 10.1016/j.celrep.2015.09.008 26456819

[B104] Varier P, Raju G, Madhusudanan P, Jerard C, Shankarappa SA (2022) A brief review of in vitro models for injury and regeneration in the peripheral nervous system. Int J Mol Sci 23:816. 10.3390/ijms23020816 PMC8775373 35055003

[B105] Vaughn LS, Lee J (2024) Neuronal injury model divulges differences in dendrite and axonal function and regeneration in adults. eNeuro 11:ENEURO.0207-24.2024. 10.1523/ENEURO.0207-24.2024 PMC11351012 39191484

[B106] von Bernhardi R, Muller KJ (1995) Repair of the central nervous system: lessons from lesions in leeches. J Neurobiol 27:353–366. 10.1002/neu.480270308 7673894

[B107] Waller TJ, Smithson LJ, Collins CA (2025) Peripheral nerve crush in Drosophila larvae. Cold Spring Harb Protoc 2025:pdb. prot108169. 10.1101/pdb.prot108169 PMC11701939 38649195

[B108] Wang H, Macagno ER (1998) A detached branch stops being recognized as self by other branches of a neuron. J Neurobiol 35:53–64. 10.1002/(SICI)1097-4695(199804)35:1<53::AID-NEU5>3.0.CO;2-A 9552166

[B109] Wang Y, Antunes M, Anderson AE, Kadrmas JL, Jacinto A, Galko MJ (2015) Integrin adhesions suppress syncytium formation in the Drosophila larval epidermis. Curr Biol 25:2215–2227. 10.1016/j.cub.2015.07.031 PMC4558245 26255846

[B110] White JS, Hua J, Su JJ, Tro KJ, Ruark EM, Hutson MS, Page-McCaw A (2026) Wound-induced syncytia outpace mononucleate neighbors during Drosophila wound repair. eLife 13:RP92593. 10.7554/eLife.92593.4 PMC13597085 42770840

[B111] Wu Y, Brock AR, Wang Y, Fujitani K, Ueda R, Galko MJ (2009) A blood-borne PDGF/VEGF-like ligand initiates wound-induced epidermal cell migration in Drosophila larvae. Curr Biol 19:1473–1477. 10.1016/j.cub.2009.07.019 PMC2783944 19646875

[B112] Yadav S, Younger SH, Zhang L, Thompson-Peer KL, Li T, Jan LY, Jan YN (2019) Glial ensheathment of the somatodendritic compartment regulates sensory neuron structure and activity. Proc Natl Acad Sci U S A 116:5126–5134. 10.1073/pnas.1814456116 PMC6421456 30804200

[B113] Yang W-K, Chien C-T (2019) Beyond being innervated: the epidermis actively shapes sensory dendritic patterning. Open Biol 9:180257. 10.1098/rsob.180257 PMC6451362 30914004

[B114] Yasothornsrikul S, Davis WJ, Cramer G, Kimbrell DA, Dearolf CR (1997) Viking: identification and characterization of a second type IV collagen in Drosophila. Gene 198:17–25. 10.1016/S0378-1119(97)00274-6 9370260

[B115] Yasunaga K-i, Kanamori T, Morikawa R, Suzuki E, Emoto K (2010) Dendrite reshaping of adult Drosophila sensory neurons requires matrix metalloproteinase-mediated modification of the basement membranes. Dev Cell 18:621–632. 10.1016/j.devcel.2010.02.010 20412776

[B116] Yip PK, Hasan S, Liu Z-H, Uff CE (2022) Characterisation of severe traumatic brain injury severity from fresh cerebral biopsy of living patients: an immunohistochemical study. Biomedicines 10:518. 10.3390/biomedicines10030518 PMC8945429 35327320

[B117] Yong VW (2005) Metalloproteinases: mediators of pathology and regeneration in the CNS. Nat Rev Neurosci 6:931–944. 10.1038/nrn1807 16288297

[B118] Yoshino J, Mali SS, Williams CR, Morita T, Emerson CE, Arp CJ, Miller SE, Yin C, Hemmi C, Motoyoshi M (2025) Drosophila epidermal cells are intrinsically mechanosensitive and modulate nociceptive behavioral outputs. eLife 13:RP95379. 10.7554/eLife.95379 PMC12068870 40353351

[B119] Zhao Z, Chen S, Luo Y, Li J, Badea S, Ren C, Wu W (2017) Time-lapse changes of in vivo injured neuronal substructures in the central nervous system after low energy two-photon nanosurgery. Neural Regen Res 12:751–756. 10.4103/1673-5374.206644 PMC5461611 28616030

