# Supplementary material for: Epidermal and ECM Damage Following Pinch Injury Restricts Dendrite Regeneration in Drosophila
Source: eNeuro. 2026 Sep 25;13(9):ENEURO.0058-26.2026. doi: 10.1523/ENEURO.0058-26.2026 (PMC13628638; doi:10.1523/ENEURO.0058-26.2026)
Supplement: Supplementary file 1 [file eneuro-13-ENEURO.0058-26.2026-s017.pdf]

FIGURE	TEST					
**Figure 1E**	**Unpaired t test**					
	P value	0.0009				
	P value summary	***				
	Significantly different (P < 0.05)?	Yes				
	One- or two-tailed P value?	Two-tailed				
	t, df	t=33.47, df=2				
**Figure 2B**						
Number of Branch Tips	**Paired t test**					
	P value	0.0004				
	P value summary	***				
	Significantly different (P < 0.05)?	Yes				
	One- or two-tailed P value?	Two-tailed				
	t, df	t=5.372, df=9				
	Number of pairs	10				
Total Branch Length	**Paired t test**					
	P value	<0.0001				
	P value summary	****				
	Significantly different (P < 0.05)?	Yes				
	One- or two-tailed P value?	Two-tailed				
	t, df	t=6.710, df=9				
	Number of pairs	10				
**Figure 2C**						
Number of Branch Tips	**Paired t test**					
	P value	0.0003				
	P value summary	***				
	Significantly different (P < 0.05)?	Yes				
	One- or two-tailed P value?	Two-tailed				
	t, df	t=5.503, df=10				
	Number of pairs	11				
Total Branch Length	**Paired t test**					
	P value	<0.0001				
	P value summary	****				
	Significantly different (P < 0.05)?	Yes				
	One- or two-tailed P value?	Two-tailed				
	t, df	t=10.31, df=10				
	Number of pairs	11				

**Figure 2D**	**Unpaired t test with Welch's correction**			**F test to compare variances**		
	P value	0.0494		F, DFn, Dfd	12.72, 10, 9	
	P value summary	*		P value	0.0007	
	Significantly different (P < 0.05)?	Yes		P value summary	***	
	One- or two-tailed P value?	Two-tailed		Significantly different (P < 0.05)?	Yes	
	Welch-corrected t, df	t=2.192, df=11.71				
**Figure 2F**						
Number of Branch Tips	**Paired t test**					
	P value	0.2876				
	P value summary	ns				
	Significantly different (P < 0.05)?	No				
	One- or two-tailed P value?	Two-tailed				
	t, df	t=1.109, df=13				
	Number of pairs	14				
Total Branch Length	**Paired t test**					
	P value	0.0064				
	P value summary	**				
	Significantly different (P < 0.05)?	Yes				
	One- or two-tailed P value?	Two-tailed				
	t, df	t=3.243, df=13				
	Number of pairs	14				
**Figure 2G**						
Number of Branch Tips	**Paired t test**					
	P value	0.0152				
	P value summary	*				
	Significantly different (P < 0.05)?	Yes				
	One- or two-tailed P value?	Two-tailed				
	t, df	t=2.739, df=15				
	Number of pairs	16				
Total Branch Length	**Paired t test**					
	P value	0.0103				
	P value summary	*				
	Significantly different (P < 0.05)?	Yes				
	One- or two-tailed P value?	Two-tailed				
	t, df	t=2.931, df=15				
	Number of pairs	16				
**Figure 2H**	**Unpaired t test with Welch's correction**			**F test to compare variances**		

	P value	0.0468		F, DFn, Dfd	18.81, 15, 13	
	P value summary	*		P value	<0.0001	
	Significantly different (P < 0.05)?	Yes		P value summary	****	
	One- or two-tailed P value?	Two-tailed		Significantly different (P < 0.05)?	Yes	
	Welch-corrected t, df	t=2.146, df=16.81				
**Figure 3C**	**Two-way ANOVA**	Ordinary				
Number of Branch Tips	Alpha	0.05				
	Source of Variation	% of total variation	P value	P value summary	Significant?	
	Row Factor	19.48	0.0864	ns	No	
	Column Factor	52.04	<0.0001	****	Yes	
	ANOVA table	SS (Type III)	DF	MS	F (DFn, DFd)	P value
	Row Factor	132669	12	11056	F (12, 32) = 1.824	P=0.0864
	Column Factor	354416	3	118139	F (3, 32) = 19.49	P<0.0001
	Residual	193965	32	6061		
	**Holm-Šídák's multiple comparisons test**	Mean Diff.	Below threshold?	Summary	Adjusted P Value	
	Pinch 24 hrs AI vs. 2p 24 hrs AI	-25.84	No	ns	0.6787	
	Pinch 24 hrs AI vs. Pinch 72 hrs AI	-197.9	Yes	****	<0.0001	
	Pinch 24 hrs AI vs. 2p 72 hrs AI	-172.1	Yes	****	<0.0001	
	2p 24 hrs AI vs. Pinch 72 hrs AI	-172.1	Yes	****	<0.0001	
	2p 24 hrs AI vs. 2p 72 hrs AI	-146.2	Yes	***	0.0001	
	Pinch 72 hrs AI vs. 2p 72 hrs AI	25.84	No	ns	0.6787	
Total Branch Length	**Two-way ANOVA**	Ordinary				
	Alpha	0.05				
	Source of Variation	% of total variation	P value	P value summary	Significant?	
	Row Factor	13.27	0.2067	ns	No	
	Column Factor	61.43	<0.0001	****	Yes	
	ANOVA table	SS (Type III)	DF	MS	F (DFn, DFd)	P value
	Row Factor	116147422	12	9678952	F (12, 32) = 1.422	P=0.2067
	Column Factor	537714738	3	179238246	F (3, 32) = 26.34	P<0.0001
	Residual	217777592	32	6805550		
	**Holm-Šídák's multiple comparisons test**	Mean Diff.	Below threshold?	Summary	Adjusted P Value	
	Pinch 24 hrs AI vs. 2p 24 hrs AI	-1166	No	ns	0.5003	
	Pinch 24 hrs AI vs. Pinch 72 hrs AI	-7107	Yes	****	<0.0001	
	Pinch 24 hrs AI vs. 2p 72 hrs AI	-7416	Yes	****	<0.0001	
	2p 24 hrs AI vs. Pinch 72 hrs AI	-5941	Yes	****	<0.0001	
	2p 24 hrs AI vs. 2p 72 hrs AI	-6251	Yes	****	<0.0001	
	Pinch 72 hrs AI vs. 2p 72 hrs AI	-309.9	No	ns	0.7782	
**Figure 3D**	**Unpaired t test with Welch's correction**			**F test to compare variances**		
	P value	0.0044		F, DFn, Dfd	3.178, 12, 10	

	P value summary	**		P value	0.0764	
	Significantly different (P < 0.05)?	Yes		P value summary	ns	
	One- or two-tailed P value?	Two-tailed		Significantly different (P < 0.05)?	No	
	Welch-corrected t, df	t=3.226, df=19.37				
**Figure 3G**						
Number of Branch Tips, 24 hrs AI	**Unpaired t test**			**F test to compare variances**		
	P value	0.0656		F, DFn, Dfd	1.293, 8, 8	
	P value summary	ns		P value	0.7248	
	Significantly different (P < 0.05)?	No		P value summary	ns	
	One- or two-tailed P value?	Two-tailed		Significantly different (P < 0.05)?	No	
	t, df	t=1.976, df=16				
Number of Branch Tips, 72 hrs AI	**Unpaired t test**			**F test to compare variances**		
	P value	0.0334		F, DFn, Dfd	1.345, 8, 8	
	P value summary	*		P value	0.6852	
	Significantly different (P < 0.05)?	Yes		P value summary	ns	
	One- or two-tailed P value?	Two-tailed		Significantly different (P < 0.05)?	No	
	t, df	t=2.327, df=16				
Total Branch Length, 24 hrs AI	**Unpaired t test**			**F test to compare variances**		
	P value	0.2552		F, DFn, Dfd	1.391, 8, 8	
	P value summary	ns		P value	0.6517	
	Significantly different (P < 0.05)?	No		P value summary	ns	
	One- or two-tailed P value?	Two-tailed		Significantly different (P < 0.05)?	No	
	t, df	t=1.180, df=16				
Total Branch Length, 72 hrs AI	**Unpaired t test**			**F test to compare variances**		
	P value	0.0002		F, DFn, Dfd	2.604, 8, 8	
	P value summary	***		P value	0.1975	
	Significantly different (P < 0.05)?	Yes		P value summary	ns	
	One- or two-tailed P value?	Two-tailed		Significantly different (P < 0.05)?	No	
	t, df	t=4.720, df=16				
**Figure 3H**						
24 hrs AI	**Unpaired t test**			**F test to compare variances**		
	P value	0.0716		F, DFn, Dfd	1.611, 8, 8	
	P value summary	ns		P value	0.5153	
	Significantly different (P < 0.05)?	No		P value summary	ns	
	One- or two-tailed P value?	Two-tailed		Significantly different (P < 0.05)?	No	

	t, df	t=1.930, df=16				
72 hrs AI	**Unpaired t test**			**F test to compare variances**		
	P value	0.0004		F, DFn, Dfd	2.298, 8, 8	
	P value summary	***		P value	0.2605	
	Significantly different (P < 0.05)?	Yes		P value summary	ns	
	One- or two-tailed P value?	Two-tailed		Significantly different (P < 0.05)?	No	
	t, df	t=4.463, df=16				
**Figure 4D**	**Two-way ANOVA**	Ordinary				
	Alpha	0.05				
	Source of Variation	% of total variation	P value	P value summary	Significant?	
	Interaction	41.94	<0.0001	****	Yes	
	Row Factor	35.65	<0.0001	****	Yes	
	Column Factor	17.55	<0.0001	****	Yes	
	ANOVA table	SS (Type III)	DF	MS	F (DFn, DFd)	P value
	Interaction	4.946	3	1.649	F (3, 38) = 22.00	P<0.0001
	Row Factor	4.204	3	1.401	F (3, 38) = 18.70	P<0.0001
	Column Factor	2.07	1	2.07	F (1, 38) = 27.62	P<0.0001
	Residual	2.847	38	0.07493		
	**Holm-Šídák's multiple comparisons test**	Predicted (LS) mean diff.	Below threshold?	Summary	Adjusted P Value	
	Pinch Spared					
	24 hrs AI vs. 72 hrs AI	-0.3421	No	ns	0.0534	
	Pinch Injured					
	24 hrs AI vs. 72 hrs AI	-1.571	Yes	****	<0.0001	
	2p "Spared"					
	24 hrs AI vs. 72 hrs AI	0.05744	No	ns	0.7183	
	2p "Injured"					
	24 hrs AI vs. 72 hrs AI	0.1223	No	ns	0.4438	
	24 hrs AI					
	Pinch Spared vs. Pinch Injured	0.001764	No	ns	0.9971	
	Pinch Spared vs. 2p "Spared"	-0.05886	No	ns	0.9971	
	Pinch Spared vs. 2p "Injured"	-0.07406	No	ns	0.9971	
	Pinch Injured vs. 2p "Spared"	-0.06062	No	ns	0.9971	
	Pinch Injured vs. 2p "Injured"	-0.07583	No	ns	0.9971	
	2p "Spared" vs. 2p "Injured"	-0.0152	No	ns	0.9971	
	72 hrs AI					
	Pinch Spared vs. Pinch Injured	-1.228	Yes	****	<0.0001	
	Pinch Spared vs. 2p "Spared"	0.3407	No	ns	0.1189	
	Pinch Spared vs. 2p "Injured"	0.3903	No	ns	0.0965	
	Pinch Injured vs. 2p "Spared"	1.568	Yes	****	<0.0001	
	Pinch Injured vs. 2p "Injured"	1.618	Yes	****	<0.0001	
	2p "Spared" vs. 2p "Injured"	0.04966	No	ns	0.7551	

**Figure 4E**	**Two-way ANOVA**					
	F	16.4				
	P value	<0.0001				
	P value summary	****				
	Significant diff. among means (P < 0.05)?	Yes				
	R squared	0.8039				
	ANOVA table	SS	DF	MS	F (DFn, DFd)	P value
	Treatment (between columns)	2051	5	410.2	F (5, 20) = 16.40	P<0.0001
	Residual (within columns)	500.4	20	25.02		
	Total	2551	25			
	**Holm-Šídák's multiple comparisons test**	Mean Diff.	Below threshold?	Summary	Adjusted P Value	
	UI Control vs. Pinch	21.05	Yes	****	<0.0001	
	UI Control vs. 2p	5.8	No	ns	0.1526	
	UI Control vs. Pinch	21.2	Yes	****	<0.0001	
	UI Control vs. 2p	6.2	No	ns	0.1526	
**Figure 1-1A**	**Unpaired t test**			**F test to compare variances**		
	P value	0.0001		F, DFn, Dfd	4.384, 2, 2	
	P value summary	***		P value	0.3714	
	Significantly different (P < 0.05)?	Yes		P value summary	ns	
	One- or two-tailed P value?	Two-tailed		Significantly different (P < 0.05)?	No	
	t, df	t=14.87, df=4				
**Figure 3-1B**						
Number of Branch Tips	**Paired t test**					
	P value	0.0071				
	P value summary	**				
	Significantly different (P < 0.05)?	Yes				
	One- or two-tailed P value?	Two-tailed				
	t, df	t=3.296, df=11				
	Number of pairs	12				
Total Branch Length	**Paired t test**					
	P value	0.0001				
	P value summary	***				
	Significantly different (P < 0.05)?	Yes				
	One- or two-tailed P value?	Two-tailed				
	t, df	t=5.693, df=11				
	Number of pairs	12				
**Figure 3-1C**						

Number of Branch Tips	**Paired t test**					
	P value	<0.0001				
	P value summary	****				
	Significantly different (P < 0.05)?	Yes				
	One- or two-tailed P value?	Two-tailed				
	t, df	t=9.485, df=12				
	Number of pairs	13				
Total Branch Length	**Paired t test**					
	P value	<0.0001				
	P value summary	****				
	Significantly different (P < 0.05)?	Yes				
	One- or two-tailed P value?	Two-tailed				
	t, df	t=11.84, df=12				
	Number of pairs	13				
**Figure 3-1D**	**Unpaired t test with Welch's correction**			**F test to compare variances**		
	P value	0.0153		F, DFn, Dfd	2.093, 12, 11	
	P value summary	*		P value	0.2317	
	Significantly different (P < 0.05)?	Yes		P value summary	ns	
	One- or two-tailed P value?	Two-tailed		Significantly different (P < 0.05)?	No	
	Welch-corrected t, df	t=2.637, df=21.39				
**Figure 4-2E**	**Two-way ANOVA**	Ordinary				
	Alpha	0.05				
	Source of Variation	% of total variation	P value	P value summary	Significant?	
	Interaction	33.06	<0.0001	****	Yes	
	Row Factor	38.28	<0.0001	****	Yes	
	Column Factor	16.69	<0.0001	****	Yes	
	ANOVA table	SS (Type III)	DF	MS	F (DFn, DFd)	P value
	Interaction	3.905	3	1.302	F (3, 30) = 15.56	P<0.0001
	Row Factor	4.522	3	1.507	F (3, 30) = 18.01	P<0.0001
	Column Factor	1.972	1	1.972	F (1, 30) = 23.56	P<0.0001
	Residual	2.511	30	0.08369		
	**Holm-Šidák's multiple comparisons test**	Predicted (LS) mean diff.	Below threshold?	Summary	Adjusted P Value	
	UI Control "Spared"					
	24 hrs AI vs. 72 hrs AI	0.01477	No	ns	0.9447	
	UI Control "Injured"					
	24 hrs AI vs. 72 hrs AI	-0.01239	No	ns	0.9536	
	Pinch Spared					
	24 hrs AI vs. 72 hrs AI	-0.3421	No	ns	0.0689	
	Pinch Injured					

	24 hrs AI vs. 72 hrs AI	-1.571	Yes	****	<0.0001	
	24 hrs AI					
	UI Control "Spared" vs. UI Control " Injured"	0.05624	No	ns	0.9967	
	UI Control "Spared" vs. Pinch Spar ed	-0.0428	No	ns	0.9967	
	UI Control "Spared" vs. Pinch Injur ed	-0.04103	No	ns	0.9967	
	UI Control "Injured" vs. Pinch Spar ed	-0.09904	No	ns	0.993	
	UI Control "Injured" vs. Pinch Injure d	-0.09727	No	ns	0.993	
	Pinch Spared vs. Pinch Injured	0.001764	No	ns	0.9967	
	72 hrs AI					
	UI Control "Spared" vs. UI Contro l "Injured"	0.02908	No	ns	0.9028	
	UI Control "Spared" vs. Pinch Sp ared	-0.3997	No	ns	0.1741	
	UI Control "Spared" vs. Pinch Inj ured	-1.627	Yes	****	<0.0001	
	UI Control "Injured" vs. Pinch Sp ared	-0.4287	No	ns	0.1741	
	UI Control "Injured" vs. Pinch Inj ured	-1.656	Yes	****	<0.0001	
	Pinch Spared vs. Pinch Injured	-1.228	Yes	****	<0.0001	
**Figure 4-2G**	**Two-way RM ANOVA**	Matching: Across ro w				
	Assume sphericity?	Yes				
	Alpha	0.05				
	Source of Variation	% of total variation	P value	P value summary	Significant?	
	Row Factor x Time	4.504	0.5297	ns	No	
	Row Factor	11.36	0.434	ns	No	
	Time	0.6667	0.568	ns	No	
	Subject	54.61	0.1034	ns	No	
	ANOVA table	SS	DF	MS	F (DFn, DFd)	P value
	Row Factor x Time	0.04363	3	0.01454	F (3, 14) = 0. 7700	P=0.52 97
	Row Factor	0.1101	3	0.03669	F (3, 14) = 0. 9710	P=0.43 40
	Time	0.006457	1	0.006457	F (1, 14) = 0. 3419	P=0.56 80
	Subject	0.529	14	0.03778	F (14, 14) = 2 .001	P=0.10 34
	Residual	0.2644	14	0.01889		
	**Holm-Šidák's multiple comparisons test**	Predicted (LS) mea n diff.	Below thresh old?	Summary	Adjusted P V alue	
	UI Control "Spared"					
	24 hrs AI vs. 72 hrs AI	-0.08168	No	ns	0.4786	
	UI Control "Injured"					
	24 hrs AI vs. 72 hrs AI	0.01558	No	ns	0.8915	
	2p "Spared"					
	24 hrs AI vs. 72 hrs AI	0.05744	No	ns	0.481	
	2p "Injured"					
	24 hrs AI vs. 72 hrs AI	0.1223	No	ns	0.1455	
	24 hrs AI					
	UI Control "Spared" vs. UI Control "Injured"	-0.0406	No	ns	0.947	

	UI Control "Spared" vs. 2p "Spared"	-0.1904	No	ns	0.475	
	UI Control "Spared" vs. 2p "Injured"	-0.2056	No	ns	0.451	
	UI Control "Injured" vs. 2p "Spared"	-0.1498	No	ns	0.5404	
	UI Control "Injured" vs. 2p "Injured"	-0.165	No	ns	0.5404	
	2p "Spared" vs. 2p "Injured"	-0.0152	No	ns	0.947	
	72 hrs AI					
	UI Control "Spared" vs. UI Control "Injured"	0.05666	No	ns	0.9914	
	UI Control "Spared" vs. 2p "Spared"	-0.05128	No	ns	0.9914	
	UI Control "Spared" vs. 2p "Injured"	-0.001612	No	ns	0.9914	
	UI Control "Injured" vs. 2p "Spared"	-0.1079	No	ns	0.9388	
	UI Control "Injured" vs. 2p "Injured"	-0.05828	No	ns	0.9914	
	2p "Spared" vs. 2p "Injured"	0.04966	No	ns	0.9914	
**Figure 4-3B**	**Paired t test**					
	P value	0.3046				
	P value summary	ns				
	Significantly different (P < 0.05)?	No				
	One- or two-tailed P value?	Two-tailed				
	t, df	t=1.177, df=4				
	Number of pairs	5				
**Figure 4-3D**	**Paired t test**					
	P value	0.391				
	P value summary	ns				
	Significantly different (P < 0.05)?	No				
	One- or two-tailed P value?	Two-tailed				
	t, df	t=1.000, df=3				
	Number of pairs	4				
**Figure 4-3E**	**Paired t test**					
	P value	0.1817				
	P value summary	ns				
	Significantly different (P < 0.05)?	No				
	One- or two-tailed P value?	Two-tailed				
	t, df	t=1.732, df=3				
	Number of pairs	4				
